# Predicting compounds that interact with the 2 known agonist-induced conformations of the human *β*1-adrenoceptor

**DOI:** 10.1016/j.molpha.2025.100081

**Published:** 2025-10-01

**Authors:** Jillian G. Baker, Victor Jun Yu Lim, Richard G.W. Proudman, Franziska N.Z. Giese, Peter Kolb

**Affiliations:** 1 Cell Signalling, School of Life Sciences, C Floor Medical School, Queen’s Medical Centre, University of Nottingham, Nottingham, UK; 2 Respiratory Medicine, Sherwood Forest Hospitals NHS Trust, King’s Mill Hospital, Nottinghamshire, UK; 3 Respiratory Medicine, Nottingham University NHS Trust, Queen’s Medical Centre, Nottingham, UK; 4 Philipps-Universität Marburg, Department of Pharmacy, Institute of Pharmaceutical Chemistry, Marburg, Germany

**Keywords:** *β*1-adrenoceptor, Secondary site, Molecular modeling, Molecular pharmacology, Affinity, Efficacy

## Abstract

The *β*1-adrenoceptor exists in at least 2 agonist-stabilized conformational ensembles: a “catecholamine” ensemble induced via the intrahelical binding site through which catecholamines and most agonists act and a “secondary” ensemble of conformations through which CGP12177 stimulates agonist responses. Several *β*-ligands stimulate agonist responses through both conformations, resulting in biphasic concentration responses, but little is known about the structure-activity relationship of such ligands. Using a structure-activity hypothesis built on the predicted poses CGP12177 and 3 biphasic agonists (alprenolol, oxprenolol, and bucindolol), predictions based on ligand similarity and structural compatibility reasoning were made about 11 other *β*1-ligands not yet tested for secondary conformation interaction and examined in radioligand binding and functional assays using human *β*1- and *β*2-adrenoceptors. Although the predictions matched with pharmacology in only 6/11 of cases, 3 novel compounds were found to induce an active-state secondary conformation. A CGP12177 derivative (methyl-pyrrole replacing the cyclic urea motif) retained catecholamine site antagonism with secondary site activation. Carteolol (related to CGP12177) and bunitrolol (similar to alprenolol) activated both conformations with biphasic concentration responses. Bunolol (CGP12177 derivative lacking nitrogen in the bicyclic system), as predicted, was a neutral antagonist with no secondary site activation. Moprolol and some bucindolol analogs appeared as conventional agonists, whereas other alprenolol and bucindolol analogs lost all receptor interaction. In a *β*1-adrenoceptor mutant (*β*1-V189T-L195Q-W199Y) where secondary site CGP12177 and pindolol interaction is lost, the 3 novel secondary-site compounds were also no longer able to stimulate secondary conformation responses, suggesting that there is a common TM4 secondary conformation-inducing interaction site.

**Significance Statement:**

The *β*1-adrenoceptor exists in 2 agonist-stabilized, pharmacologically distinguishable conformations. This study pinpointed the interaction site through which the alternative conformation is stabilized and suggested and evaluated additional ligands, thus providing possible molecular determinants.

## 1 Introduction

The agonist actions of several *β*1-adrenoceptor (AR) ligands cannot be explained by interactions at a single site or conformation.^1^ Low concentrations of certain *β*-AR ligands (eg, pindolol^2^ and CGP12177^3^) block catecholamine responses (via the catecholamine conformation), whereas higher concentrations stimulate partial agonist responses via a secondary site.^4^, ^5^, ^6^ Furthermore, catecholamine conformation full and partial agonist responses are readily inhibited by *β*-antagonists, whereas secondary conformation agonist responses are relatively resistant to inhibition.^5^^,^^7^^,^^8^ In cat, rodent, and human myocardium, pindolol stimulates biphasic agonist responses.^9^^,^^10^ Studies with cloned receptors and knockout animals confirmed that all responses are occurring via *β*1-ARs.^11^, ^12^, ^13^, ^14^, ^15^, ^16^, ^17^, ^18^, ^19^, ^20^, ^21^ Thus, the *β*1-AR exists in at least 2 agonist-stabilized pharmacological conformations and there are 4 measurable pharmacological phenomena that allow the additional *β*1-secondary conformation to be distinguished from the conventional *β*1-catecholamine conformation: (A) in the same system (cells, tissues, or whole animals), secondary conformation agonist responses occur at substantially higher concentrations (ie, with lower affinity) than the concentration required to bind to the catecholamine conformation (ie, EC_50_ > K_D,_ where K_D_ can be measured from radioligand binding or inhibition of agonist responses); (B) inhibition of secondary conformation agonist responses requires substantially higher concentrations of *β*-antagonist than inhibition of catecholamine-conformation agonist responses; (C) some ligands stimulate biphasic agonist responses where the first component is readily inhibited and the second component relatively resistant to *β*-antagonism; (D) as to date all secondary conformation agonists bind to the catecholamine conformation with higher affinity (point (A) above), in the presence of a catecholamine site agonist, increasing concentrations of secondary conformation agonist inhibit the catecholamine response at low concentrations and then stimulate their own agonist response at higher concentrations, creating a dip in the curve not reconcilable with a single site of action.

The human *β*3-AR also has a secondary conformation,^22^ whereas to date there is no pharmacological evidence of a *β*2-AR secondary conformation.^23^, ^24^, ^25^

We know several things about *β*1-AR secondary conformations. First, several ligands stabilize active-state secondary conformations, including alprenolol, bucindolol, carazolol, carvedilol, CGP12177, cyanopindolol, LY362884, oxprenolol, pindolol, SDZ21009, and SR59230A.^5^^,^^7^^,^^8^^,^^10^^,^^11^^,^^15^^,^^16^^,^^19^^,^^21^^,^^24^^,^^26^^,^^27^ Second, *β*1-AR secondary conformations exist in animal and human heart and blood vessels (see references above), so although the physiological relevance remains unknown, it is a genuine pharmacological phenomenon. Third, carvedilol therapeutic human plasma concentration (eg, for heart failure and ischemic heart disease) is 100 ng/mL (300 nM^28^), a concentration at which carvedilol stabilizes a secondary conformation.^29^ Fourth, although *β*-blockers inhibit agonist responses at both conformations (albeit at different concentrations) the rank order of affinity differs, confirming the secondary conformation as a distinct entity, not a “mirror” of the catecholamine conformation.^21^ Fifthly, CGP12177 and pindolol analog studies have highlighted certain chemical moieties required for their secondary conformation agonism.^30^^,^^31^ Sixth, amino acid residues toward the extracellular end of transmembrane 4 (TM4: V189, L195, and W199, that are notably conserved between species for both *β*1- and *β*3-AR but are different in *β*-ARs where no secondary conformation has been described^25^) are required for both CGP12177 and pindolol secondary conformation stimulation suggesting that both ligands stabilize a common secondary conformation. However, it is unknown whether a plethora of “*β*1-AR secondary sites” exists, accessed by different ligands, or if there is just one “secondary site” and V189/L195/W199 are important for all secondary conformation agonists.

Little is known about the structure-activity relationship of secondary conformation ligands and even less regarding how ligands access both the catecholamine *and* secondary sites. One explanation for biphasic responses (eg, pindolol) is that at low concentrations, a molecule occupies the catecholamine site as a typical high-affinity partial agonist. Higher concentrations allow a second molecule to occupy a distinct site (with lower affinity) that directly stabilizes a different overall higher-efficacy receptor conformation. Alternatively, the second molecule could occupy a site that behaves allosterically, augmenting the catecholamine site efficacy (ie, allosterically augmenting its own efficacy, but not affinity). Both explanations hold for CGP12177, where catecholamine site occupation usually results in high-affinity neutral antagonism and secondary site occupancy either causes a direct (lower affinity) stimulation of an active-state conformation of the receptor or an allosteric augmentation of very low (undetected) catecholamine site efficacy.

Given the plethora of structural data for *β*-ARs, we investigated the nature and existence of secondary sites. Based on a structure-activity hypothesis built on the predicted poses of known secondary ligands, we predicted, then pharmacologically tested, whether other known (but untested) *β*-AR compounds would stabilize an active-state conformation and then determined whether this was via the same site as the “extra-TM4” site used by CGP12177 and pindolol.

## 2 Terminology

The observed pharmacological catecholamine and secondary conformations are, as all receptor conformational states, structurally most likely a set of closely related conformations, and will be abbreviated to “CCE” (catecholamine conformation ensemble) and “SCE” (secondary conformation ensemble). Going forward, SCE will be used for discussion of pharmacological phenomena. In contrast, for discussion of the actual site through which the SCE is stimulated, V189/L195/W199 site or known site 2 (“KS2”; see beginning of Results for an explanation of this terminology) will be used. The physical site for ligand-amino acid interactions where, eg, catecholamines bind to the *β*1-AR, will be termed “intrahelical binding site” (IBS^32^) and refers to the general cavity and not specific ligand-amino acid interactions within it, to better distinguish it from the “extra-TM4” V189/L195/W199 site.

## 3 Materials and methods

### 3.1 Materials

**Fig. 1:**
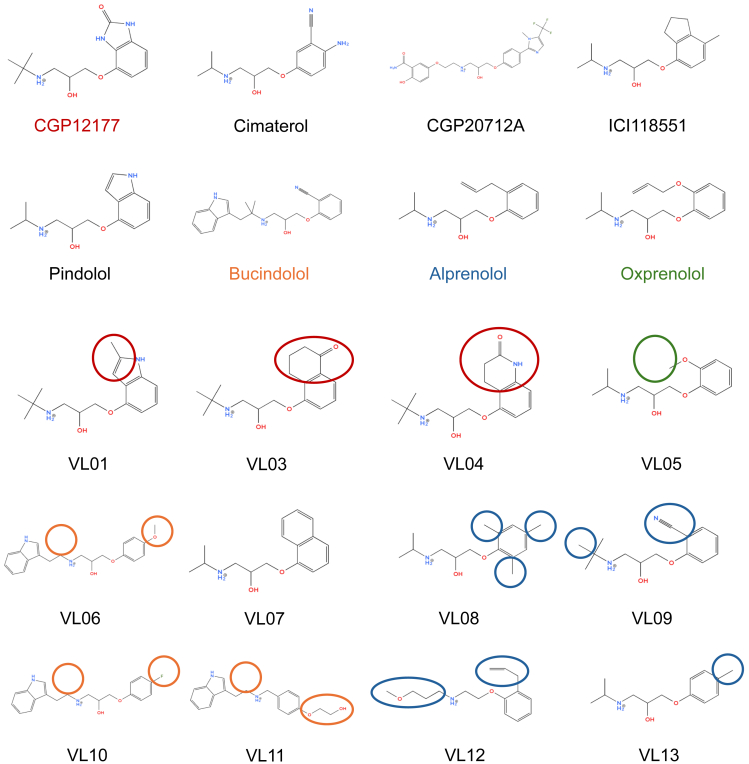
Two-dimensional chemical structures of known and predicted compounds used in this study. The VL compound chemical moieties mentioned in the *Results* and *Discussion* are highlighted with ellipses in order to make identifying the moieties easier. Ellipse colors match the font colors of the parental compounds’ names.

^3^H-CGP12177 (NET1061250UC), Steadylite Plus (luciferase detection), Microscint 20, and Ultima Gold XR scintillation fluid were from Revvity (formerly Perkin-Elmer). Cimaterol (0435), CGP20712A (1024), bucindolol (2658), and oxprenolol (3288) were from Tocris. **VL01** (Mcule-7804650113, Key Organics P-18694452), **VL03** (Mcule-5582827213, MedChemExpress P-588278446), **VL04** (Mcule-286344860, MedChemExpress P-588276645), **VL11** (Mcule-2229578802, Chembridge P-3794668), **VL12** (Mcule-4273841503, Chembridge P-6086407), and **VL13** (Mcule-9006078691, Chembridge P-5272537) were obtained via Mcule (Budapest, Hungary). **VL05** (Amb17760690), **VL06** (Amb24208310), **VL07** (Amb6342301), and **VL08** (Amb9653) were from Ambinter. **VL09** (EN300-1268038) and **VL10** (Z31380545) were from Enamine. Figure 1 shows the structures of the compounds used in this study, and Supplemental Tables 1 and 2 list their SMILES and IUPAC names, respectively. CGP12177 (C125), ICI118551 (I127), alprenolol (A8625), pindolol (P0778), and all other reagents were from Sigma Aldrich.

### 3.2 Docking calculations

Human *β*1-AR was modeled using MODELLER^33^ for both active and inactive conformations. The crystallographic structure with PDB ID 2VT4 was used as template to model the inactive conformation, whereas PDB ID 3SN6 was used for the active conformation. The G*α*s subunit was added to the *β*1-AR active-state model by superimposing this model with the ternary complex of the *β*2-AR (PDB ID 3SN6) and removing the receptor portion and small-molecule ligand of 3SN6. Swissdock^34^ was used to dock CGP12177 to the entire *β*1-AR protein complex, including the Gαs. As CGP12177 is a hydrophilic ligand and therefore unlikely to pass through the lipid bilayer, we focused on the predicted binding sites accessible from the extracellular space. CGP12177 was then manually docked to the binding pocket between TM3 and TM4 (KS2), using the docking poses from Swissdock as reference. The residues around CGP12177 were afterward minimized using the CHARMM27 forcefield in MOE2019.^35^

### 3.3 Similarity searches for analogs

Cartblanche^36^ was used to search for purchasable compounds with (1) the same substructure or (2) similarity based on the Tanimoto coefficient to known compounds with access to the SCE. The compounds were manually curated for insights into possible structure-activity relationships for the secondary conformation.

### 3.4 Cell culture

Clonal cell lines of Chinese hamster ovary (CHO)-K1 cells (RIDD: CVCL_0214) stably transfected with the wild-type human *β*1-AR and a CRE-luciferase reporter gene (CHO-*β*1-CRE-luc), cells stably transfected with the human *β*1- or *β*2-AR and CRE-SPAP reporter gene (CHO-*β*1-CRE-SPAP, CHO *β*2-CRE-SPAP), and cells expressing the CRE-SPAP reporter gene but with no transfected receptor (CHO-CRE-SPAP) were used.^19^^,^^23^ In addition, stable mixed populations of cells expressing human wild type *β*1-WT (wild type), *β*1-AR with 3 mutations (*β*1-V189T-L195Q-W199Y^25^) were also used. These were generated by transfecting a monolayer of CHO-K1 cells stably expressing a CRE-SPAP reporter in a T75 with 10 ng DNA in 100 *μ*L Lipofectamine and 8 mL OPTIMEM as per manufacturer’s instructions. Cells were selected for 3 weeks using G418 (1 mg/mL for receptor) and hygromycin (200 *μ*g/mL for CRE-SPAP reporter gene) during which time there were passaged at least twice until confluent T75 or T175 flasks of cells were obtained. Selection antibiotics were then removed.

All CHO cells were grown in Dulbecco’s modified Eagle’s medium nutrient mix F12, containing 2 mM L-glutamine and 10% fetal calf serum at 37 °C in humidified 5% CO_2_: 95% air atmosphere. With the exception of cells under active selection for the generation for the stable mixed populations, cells were grown in flasks and plates in the absence of antibiotics. Mycoplasma contamination is intermittently monitored within the laboratory (negative), but cell lines were not tested routinely with each experiment.

### 3.5 ^3^H-CGP12177 whole-cell binding

The affinity (K_D,_ concentration required to bind half of the receptors) of ^3^H-CGP12177 was determined by saturation binding (using 0.008–8.911 nM ^3^H-CGP12177 and using 10 *μ*M propranolol to determine nonspecific binding). The affinity for competing ligands was determined by incubating the competing ligand in the presence of a fixed concentration of ^3^H-CGP12177. Briefly, cells were grown to confluence in tissue-culture-treated white-sided 96-well view plates. The media was removed and nonradioligands in 100 *μ*M serum free media (sfm = Dulbecco’s modified Eagle’s medium nutrient mix F12 containing 2 mM L-glutamine) at twice final concentration were added to the wells (including 100 *μ*L 20 *μ*M propranolol to wells used to determine nonspecific binding). This was immediately followed by the addition of 100 *μ*L ^3^H-CGP12177 (thus a total volume of 200 *μ*L and a 1:2 dilution in the well), as previously described.^37^ Cells were incubated for 2 hours at 37 °C before being washed with 2 × 200 mL cold (4 °C) PBS. A white bottom was applied to convert the wells to white-bottomed, white-sided wells, 100 *μ*L Microscint 20 was then added to each well, and a clear sealant top was applied. The plates left for at least 8 hours in the dark before being counted on a Topcount for 2 minutes per well. Total binding (6 wells) and nonspecific binding (6 wells, 10 *μ*M propranolol final concentration) was determined in each plate.

### 3.6 CRE-luciferase production

Cells were plated into tissue-culture-treated white-sided 96-well plates, grown to confluence and CRE-luciferase production determined after 5 hours as previously described.^21^ Briefly, the media was removed and replaced with 100 *μ*L sfm or sfm containing a final concentration of antagonist and incubated for 15 minutes at 37 ˚C. Agonist (in 10 *μ*L sfm, at 10 times final concentration) was then added to the wells (1:10 dilution in the wells), positive control (isoprenaline final well concentration 10 *μ*M), and the plates incubated for 5 hours at 37 °C in a humidified 5% CO_2_: 95% air atmosphere. After 5 hours, all well contents were removed, a white bottom applied to the plate, 40 *μ*L luclite reagent (= 20 *μ*L Steadylite Plus + 20 *μ*L PBS containing 1 mM Ca^2+^ and 1 mM Mg^2+^) were added to each well, and the plates kept in the dark for 5 minutes before being read on a Topcount for 2 seconds per well.

### 3.7 CRE-SPAP production

Cells were plated into tissue-culture-treated clear 96-well plates, grown to confluence and CRE-SPAP production determined as previously described.^38^ Briefly, at 24 hours, the media was removed and 100 *μ*L sfm added to each well (to serum starve the cells) for a second 24 hours before experimentation. Sfm was removed from wells, and 100 *μ*L sfm or 100 *μ*L antagonist in sfm at final concentration added to the wells and the plate, incubated for 15 minutes at 37 °C. Agonist (in 10 *μ*L, at 10 times final concentration) was then added to the relevant wells (1:10 dilution in the wells), the positive control (isoprenaline, final concentration 10 *μ*M) to control wells, and the plates incubated for 5 hours at 37 °C in a humidified 5% CO_2_ : 95% air atmosphere. After 5 hours, all well contents were removed and replaced with 40 *μ*L sfm and incubated for 1 hour (37 °C in a humidified 5% CO_2_ : 95% air atmosphere). Plates were then placed in a 65 °C oven for 30 minutes to destroy all endogenous phosphatase, cooled, 100 *μ*L 5 mM pNPP in diethanolamine buffer added to each well, and read on a Dynatech MRX plate reader at 405 nm once the yellow color had started developing.

### 3.8 Data analysis

#### 3.8.1 Whole-cell binding

The K_D_ of ^3^H-CGP12177 was determined by saturation binding fitted using the nonlinear regression program GraphPad Prism 10 (GraphPad) to equation 1:

(1) $$\begin{array}{c} \text{SB}\;= \end{array}\frac{\left(A\times B_{\max}\right)}{\left(A+K_{D}\right)}$$

where A is the concentration of ^3^H-CGP12177, B_max_ is the maximal specific binding, and K_D_ is the dissociation constant of ^3^H-CGP12177.

The affinity of the other ligands was determined from competition binding. A sigmoidal concentration-response curve was then fitted to the data using GraphPad Prism 10, and the IC_50_ was determined as the concentration required to inhibit 50% of the specific binding using equation 2.

(2) $$\begin{array}{c} \%\;\text{uninhibited}\;\text{binding}\;= \end{array}\begin{array}{ccc} 100 & -\frac{\left(100\;\times \;[A]\right)}{\left([A]+{\text{IC}}_{50}\right)} & +\text{NS} \end{array}$$

where [A] is the concentration of the competing ligand, IC_50_ is the concentration at which half of the specific binding of ^3^H-CGP12177 has been inhibited, and NS is the nonspecific binding.

From the IC_50_ value and the known concentration of ^3^H-CGP12177, a K_D_ value (concentration at which half the receptors are bound by the competing ligand) was calculated using equation 3 (Cheng-Prusoff equation):

(3) KD=IC501+H3-CGP12177/KDH3-CGP12177

#### 3.8.2 CRE-luciferase and CRE-SPAP functional experiments:

Many agonist responses were best described by a one-site sigmoidal agonist concentration-response curve. Curves were fitted to the data using equation 4:

(4) $$\begin{array}{cc} \text{Response}\;= & \frac{{\text{E}}_{\text{max}}\times \left[A\right]}{{\text{EC}}_{50}+\left[A\right]} \end{array}$$

where E_max_ is the maximal response, [A] is the agonist concentration, and EC_50_ is the concentration of agonist that produces 50% of the maximal response.

Antagonist K_D_ values were then calculated from the parallel shift of the agonist concentration responses in the presence of a fixed concentration of antagonist using equation 5 (Gaddum equation):

(5) $$\begin{array}{cc} \text{DR}\;= & 1+\frac{\left[B\right]}{{\text{K}}_{\text{D}}}\; \end{array}$$

where DR (dose ratio) is the ratio of the agonist concentration required to stimulate an identical response in the presence and absence of a fixed concentration of antagonist [B].

In experiments where 3 different fixed concentrations of the same antagonist were used, Schild plots were constructed using equation (6):

(6) $$\begin{array}{cc} \log\;\left(\text{DR}-1\right)\;= & \log\left[B\right]-\log\left(K_{D}\right) \end{array}$$

These points were then fitted to a straight line. A slope of 1 then indicates competitive antagonism.^39^

Where clear partial agonism was present (eg, Fig. 3D), the partial agonist affinity was calculated by the method of Stephenson^40^ using equation 7.

(7) $$\begin{array}{ccc} K_{D}\;\text{partial}\;\text{agonist} & = & \frac{Y\times \left[P\right]}{1-Y}\\ \text{where}\;Y & = & \frac{\left[A_{2}\right]-\left[A_{1}\right]}{\;\left[A_{3}\right]} \end{array}$$

where [P] is the concentration of the partial agonist, [A_1_] is the concentration of the agonist at the point where the fixed partial agonist causes the same response, [A_2_] is the concentration of agonist causing a given response above that achieved by the partial agonist, and [A_3_] is the concentration of the agonist, in the presence of the partial agonist, causing the same stimulation as [A_2_].

Some agonist’s responses were clearly best described by a 2-site stimulatory concentration response (eg, Fig. 11B); thus equation 8 was fitted to these data:

(8) $$\begin{array}{ccc} \%\;\text{maximal}\;\text{stimulation} & = & \frac{\left[A\right]\times N\;}{\left(\left[A\right]+\text{EC}1_{50}\right)}+\frac{\left[A\right]\times \left(100-N\right)}{\left(\left[A\right]+\text{EC}2_{50}\right)} \end{array}$$

where N is the percentage of site 1, [A] is the concentration of agonist, and EC1_50_ and EC2_50_ are the respective EC_50_ values for the 2 agonist sites.

At other times, the concentration-response curves (eg, Fig. 3E) clearly contained 2 components: an inhibitory response followed by a stimulatory response; thus, a 2-site analysis was performed using equation 9:

(9) $$\text{Response}=\text{Basal}+\left(\text{control}\;-\;\text{Basal}\right)\cdot \left[1-\frac{\left[A\right]}{\left(\left[A\right]+{\text{IC}}_{50}\right)}\right]+S_{\mathrm{MAX}}\cdot \left[\frac{\left[A\right]}{\left(\left[A\right]+{\text{EC}}_{50}\right)}\right]$$

where "Basal" is the response in the absence of agonist, "control" is the response to a fixed concentration of control (eg, cimaterol 30 nM), [A] is the concentration of agonist, IC_50_ is the concentration of agonist that inhibits 50% of the response to control, EC_50_ is the concentration of agonist that caused a half maximal stimulation, and S_MAX_ is the maximum stimulation of this component.

## 4 Results

### 4.1 Molecular modeling to identify ligands with secondary-site interaction

**Fig. 2:**
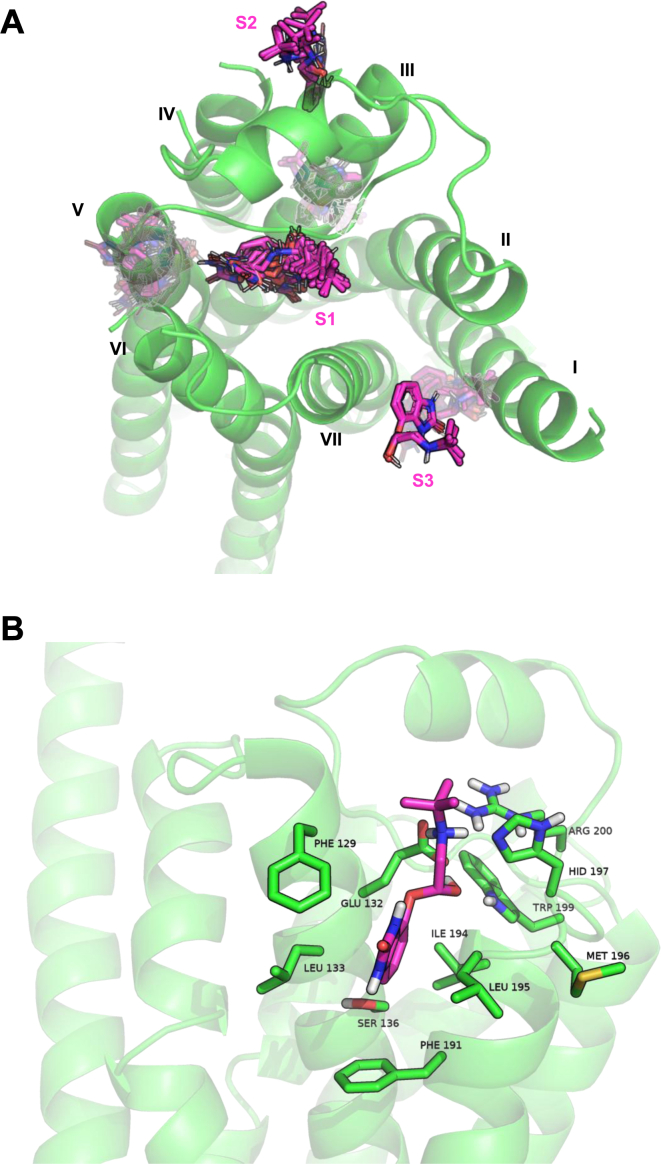
(A) Top view of the *β*1-AR (green ribbons; TMs numbered with roman numerals) and the 3 extracellular binding sites identified via Swissdock (S1–S3). Docked poses of CGP12177 are shown with purple carbons. (B) Side view of the *β*1-AR (green ribbons) toward TM4, showing the residues (sticks with green carbons) surrounding the most plausible pose of CGP12177 (purple carbons) in KS2.

After docking CGP12177 to the entire surface of the *β*1-AR, we observed 3 clusters of poses on the receptor. The classical IBS^32^ (labeled S1 in Fig. 2A), a second site was located between helices III and IV (S2 in Fig. 2A), and a third one between helices I and VII (S3 in Fig.2A). In the latter site, none of the poses of CGP12177 formed favorable interactions, and we therefore continued with the second site, which corresponds to KS2.^32^ The residues surrounding CGP12177 in KS2 are shown in Fig. 2B, and mutations of these residues are likely to affect the binding of CGP12177 to the secondary pocket. These residues are F129, E132, L133, S136, F191, I194, L195, M196, W199, H198, and R200. Of these, only L133 (TM3) and L195 and W199 (TM4) differ between the human *β*1- and *β*1-AR receptor. This is in keeping with earlier studies where L195 and W199 were found to be essential for CGP12177’s secondary conformation interaction. As modeling has suggested only this one plausible extracellular non-IBS interaction, this suggests that there is indeed likely only one non-IBS or secondary interaction site accessed by CGP12177 on the *β*1-AR, and it is KS2.

Based on 5 already known compounds that activate an SCE at the *β*1-AR (alprenolol, bucindolol, CGP12177, oxprenolol, and pindolol), we searched for analogs and retrieved 3 analogs of CGP12177, 1 analog of pindolol, 4 analogs of bucindolol, and 4 analogs of alprenolol and oxprenolol from the ZINC20 library.

The ligands were sourced and passed to the pharmacology team who analyzed the compounds blind to the predictions made by molecular modeling.

### 4.2 ^3^H-CGP1277 whole-cell binding in stable cell lines

**Fig. 3:**
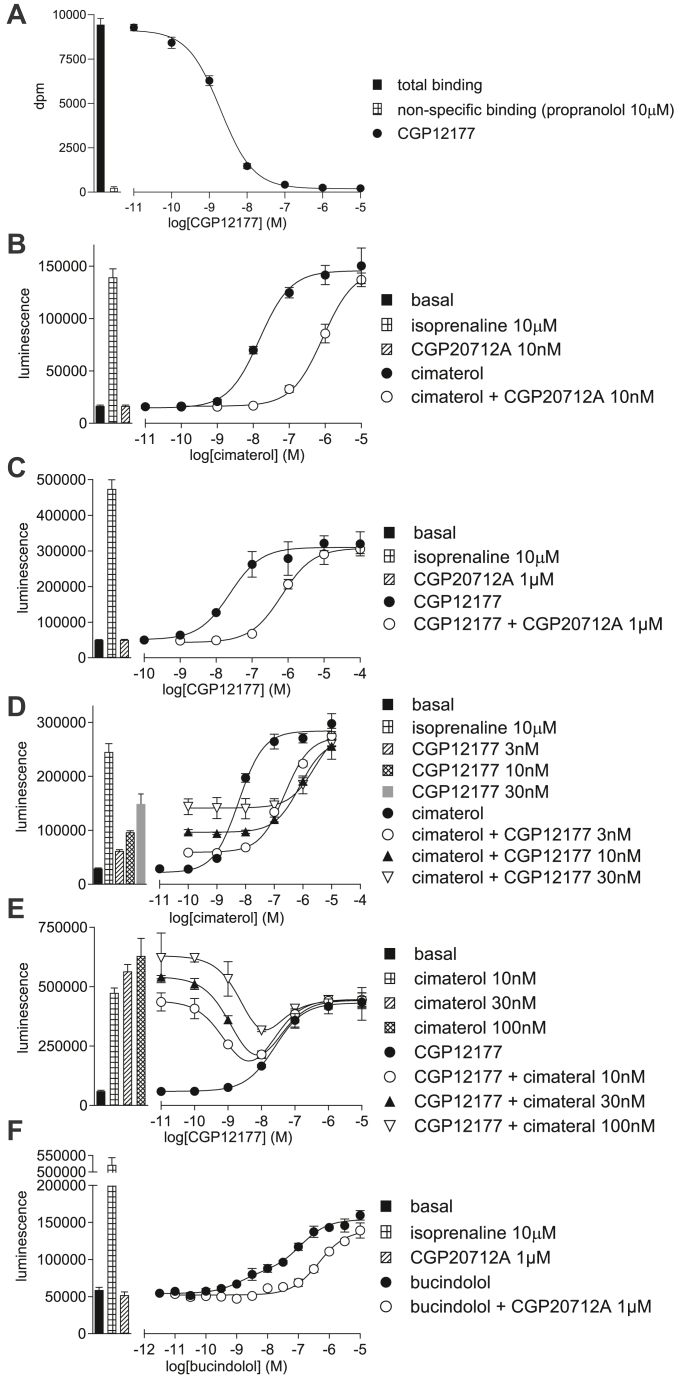
(A) Inhibition of ^3^H-CGP12177 whole-cell binding by CGP12177 in CHO-*β*1-CRE-SPAP cells. The concentration of ^3^H-CGP12177 is 0.71 nM. (B–F) CRE-luciferase production in CHO-*β*1-CRE-luciferase cells in response to (B) cimaterol in the absence and presence of 10 nM CGP20712A; (C) CGP12177 in the absence and presence of 1 *μ*M CGP20712A; (D) cimaterol in the absence and presence of 3, 10, or 30 nM CGP12177; (E) CGP12177 in the absence and presence of 10, 30, or 100 nM cimaterol; and (F) bucindolol in the absence and presence of 1μM CGP20712A. Bars represent basal CRE-luciferase production and that in response to 10 *μ*M isoprenaline and other compounds alone as listed with each graph. Data points are mean ± SD of triplicate determinations and are representative of (A) 7, (B) 10, (C) 10, (D) 6, (E) 6, and (F) 7 separate experiments.

The K_D_ value for ^3^H-CGP12177 and receptor expression levels have previously been determined in the stable cell lines as 0.15 nM (79 fmol/mg protein) in CHO-*β*1-CRE-luc cells, 0.42 nM (1146 fmol/mg protein) in CHO-*β*1-CRE-SPAP cells, and 0.17 nM (466 fmol/mg protein) in CHO-*β*2-CRE-SPAP cells.^19^^,^^23^ The binding affinity for other ligands (including non-radiolabelled CGP12177) was determined from competition binding (Table 1, Fig. 3). The high selectivity of the *β*1 antagonist CGP20712A for *β*1-AR, and the *β*2 antagonist ICI118551 for *β*2-AR, confirms the presence of the *β*1- and *β*2-AR in the CHO-*β*1-CRE-SPAP and CHO-*β*2-CRE-SPAP lines, respectively. The concentration of ^3^H-CGP12177 used in these experiments will only detect ligands binding at the high-affinity catecholamine confirmation of the human *β*1-AR; thus, Table 1 shows the affinity of these ligands for the catecholamine conformation of the *β*1-AR and does not give any information about secondary-site interaction.

**Table 1:** Affinity (log K_D_ values) of *β*-AR ligands for the *β*1 and *β*2-AR obtained from ^3^H-CGP12177 whole-cell binding to CHO-*β*1-CRE-SPAP and CHO-*β*2-CRE-SPAP cells, respectively The values are mean ± s.e.mean for n separate experiments. The *β*2 over *β*1-selectivity is also given; thus, ICI118551 has 603-fold higher affinity for the *β*2- than *β*1-adrenoceptor, whereas CGP20712A (at 0.0008-fold) has 1202-fold higher affinity for the *β*1-adrenoceptor.

Ligand	Log K_D_*β*1	n	Log K_D_*β*2	n	*β*2-Selectivity
CGP20712A	*−*8.80 ± 0.07	6	*−*5.72 ± 0.05	6	0.0008
ICI118551	*−*6.74 ± 0.06	8	*−*9.52 ± 0.06	6	603
Cimaterol	*−*6.43 ± 0.05	6	*−*7.11 ± 0.07	6	4.8
CGP12177	*−*9.29 ± 0.07	7	*−*9.75 ± 0.07	7	2.9
Alprenolol	*−*8.20 ± 0.06	6	*−*9.46 ± 0.05	6	18.2
Bucindolol	*−*9.24 ± 0.07	6	*−*10.29 ± 0.07	6	11.2
Oxprenolol	*−*8.06 ± 0.06	6	*−*9.37 ± 0.09	6	20.4
Pindolol	*−*8.71 ± 0.07	6	*−*9.52 ± 0.09	6	6.5
**VL01**	*−*9.45 ± 0.08	7	*−*10.53 ± 0.07	7	12.0
**VL03**	*−*8.29 ± 0.04	7	*−*10.09 ± 0.04	7	63.1
**VL04**	*−*8.64 ± 0.09	7	*−*9.76 ± 0.10	7	13.2
**VL05**	*−*7.62 ± 0.06	7	*−*7.91 ± 0.06	7	1.9
**VL06**	*−*5.95 ± 0.06	7	*−*6.01 ± 0.08	7	1.1
**VL07**	*−*8.21 ± 0.07	7	*−*9.19 ± 0.05	6	9.5
**VL08**	IC_50_>*−*4	5	*−*5.49 ± 0.16	5	
**VL09**	*−*8.51 ± 0.04	7	*−*8.96 ± 0.08	7	2.8
**VL10**	*−*6.73 ± 0.05	7	*−*7.05 ± 0.06	6	2.1
**VL11**	IC_50_>*−*4	5	*−*5.05 ± 0.14	5	
**VL12**	IC_50_>*−*4	5	*−*5.17 ± 0.16	5	
**VL13**	No binding to 100 *μ*M	5	No binding to 100 *μ*M	5	

### 4.3 CHO-β1-CRE-luciferase responses

#### 4.3.1 Pharmacological characterization of the CCE and SCEs with cimaterol and CGP12177

Cimaterol (log EC_50_ −8.07 ± 0.03, 106.2 ± 2.0% isoprenaline maximum, *n* = 19, Table 2) stimulated an agonist response in the CHO-*β*1-CRE-luciferase cells that was inhibited by CGP20712A with high affinity (log K_D_ CGP210712A −9.47 ± 0.12, *n* = 12; Fig. 3), and by ICI118551 with lower affinity (log K_D_ ICI118551 −7.33 ± 0.06, *n* = 5, Table 3) confirming the cimaterol response was indeed occurring via the *β*1-AR. CGP12177 also inhibited the cimaterol response as a partial agonist to yield a log K_D_ value of −9.87 ± 0.06 *n* = 11 (as calculated by partial agonist method^40^; Fig. 3). Cimaterol is an efficacious agonist, with an affinity (log K_D_ of −6.43 or 372 nM) and log EC_50_ of −8.07 (or 8.5 nM), and thus it only needs to bind a few receptors to induce a full agonist response.

**Table 2:** Agonist actions of compounds in CHO-*β*1-CRE-luciferase cells Log EC_50_ values are given, with % of the response they generated compared to 10 *μ*M isoprenaline. The values are mean ± s.e.mean for n separate experiments.

Ligand	Log EC_50_	% isop	n	Log K_D_ CGP20712A	n
Alprenolol	*−*6.49 ± 0.11	3.2 ± 0.8	8		
Bucindolol^a^	EC_50_1 *−*9.27 ± 0.14 EC_50_2 *−*7.24 ± 0.11 23.3 ± 2.4% at EC_50_1	23.7 ± 1.7	7	*−*8.06 ± 0.11	8
CGP12177	*−*7.54 ± 0.04	54.2 ± 3.5	25	*−*7.37 ± 0.09	17
Cimaterol	*−*8.07 ± 0.03	106.2 ± 2.0	19	*−*9.47 ± 0.12	12
Oxprenolol	*−*6.28 ± 0.11	5.3 ± 0.8	8		
Pindolol	*−*5.64 ± 0.12	11.7 ± 1.4	8		
**VL01**	*−*6.78 ± 0.05	19.7 ± 2.4	16	*−*7.29 ± 0.08	13
**VL03**	No response		5		
**VL04**	*−*6.12 ± 0.06	23.7 ± 3.1	13	*−*7.39 ± 0.06	22
**VL05**	*−*7.19 ± 0.08	9.7 ± 0.8	18	*−*9.54 ± 0.11	15
**VL06**	*−*5.85 ± 0.31	2.7 ± 0.8	8		
**VL07**	No response		5		
**VL08**	No response		5		
**VL09**	*−*6.89 ± 0.03	23.9 ± 2.2	17	*−*7.84 ± 0.06	18
**VL10**	*−*6.81 ± 0.06	13.8 ± 1.5	20	*−*9.73 ± 0.10	17
**VL11**	No response		5		
**VL12**	No response		5		
**VL13**	No response		5		

a Bucindolol stimulated a biphasic concentration-response curve (Fig. 3F). The log EC_50_ at both components are given, along with % occurring via EC_50_1 and % of isoprenaline of the whole response. The K_D_ value for CGP20712A is for inhibition of the first component.

**Table 3:** Log K_D_ values of ligands determined from a rightward shift of agonist responses to cimaterol or CGP12177 in CHO-*β*1-CRE-luciferase cells Values are calculated using the method of Gaddum (eq. 5). The values are mean ± s.e.mean for n separate experiments. The ratio of affinities for the 2 conformations is also shown. Compounds all have higher affinity for the catecholamine-induced than secondary conformation, eg, VL01 has log 3.55 (ie, 3500-fold) high affinity for the catecholamine rather than secondary conformation, whereas VL10 has log 1.64 (44-fold) higher affinity for the catecholamine site. As in^21^, this corroborates that the secondary conformation is a distinct entity (not a low-affinity “mirror” of the catecholamine conformation), with compounds interacting with it in a distinct manner with both distinct affinity (measured here) and efficacy (previous table).

	Catecholamine Conformation		Secondary Conformation		Log ratio of affinities
	Cimaterol as agonist		CGP12177 as agonist		
	Log K_D_	n	Log K_D_	n	
CGP12177	*−*9.87 ± 0.06^a^	11			
CGP20712A	*−*9.47 ± 0.12	12	*−*7.37 ± 0.09	17	2.10
ICI118551	*−*7.33 ± 0.06	5	*−*5.76 ± 0.05	6	1.57
**VL01**	*−*10.19 ± 0.05	12	*−*6.64 ± 0.07^a^	7	3.55
**VL03**	*−*8.91 ± 0.05	6	*−*6.38 ± 0.01	6	2.53
**VL04**	*−*9.12 ± 0.09	8	*−*5.70 ± 0.10∗	8	3.42
**VL05**	*−*8.00 ± 0.04^a^	8	No shift 10 *μ*M	5	>3
**VL06**	*−*6.46 ± 0.04	5	No shift 10 *μ*M	5	>1.46
**VL07**	*−*8.87 ± 0.06	6	*−*6.16 ± 0.04	9	2.71
**VL08**	<*−*5	5	No shift 10 *μ*M	5	
**VL09**	*−*8.84 ± 0.03^a^	6	−6.03 ± 0.10^a^	7	2.81
**VL10**	*−*7.21 ± 0.03^a^	7	−5.57 ± 0.10^a^	7	1.64
**VL11**	<*−*5	6	No shift 10 *μ*M	5	
**VL12**	No shift at 10 *μ*M	5	No shift 10 *μ*M	5	
**VL13**	No shift at 10 *μ*M	6	No shift 10 *μ*M	5	

a Ligand with partial agonism and therefore the K_D_ was calculated using the method of Stephenson (eq. 7).

CGP12177 stimulated a partial agonist response (log EC_50_ −7.54 ± 0.04, 54.2 ± 3.5% isoprenaline maximum, *n* = 25; Fig. 3, Table 2) that required higher concentrations of CGP20712A and ICI118551 to inhibit and thus yielded log K_D_ values of −7.37 ± 0.09 (*n* = 17) and −5.76 ± 0.05 (*n* = 6), respectively (Table 3). These values are very similar to previous data.^21^ Thus, within this cell line, the affinity of CGP12177 as determined from inhibition of an agonist (log K_D_ −9.87 or 0.13 nM) or as determined from binding (0.42 nM from saturation binding and log K_D_ −9.29 or 0.51 nM, from competition binding) is at odds with its EC_50_ (log EC_50_ −7.54 or 29 nM). For a partial agonist activating a single conformation the K_D_ and EC_50_ should be the same. However, here when half the receptors are bound (0.13 nM), there is no stimulatory response, yet 29 nM stimulates half the maximum response obtainable with CGP12177, suggesting that the high-affinity binding of CGP12177 and the stimulatory response are acting at 2 different sites of the receptor (evidence (A) for SCE activation as outlined in the Introduction). Furthermore, the concentrations of antagonists (CGP20712A and ICI118551) required to inhibit the CGP12177 agonist response were far greater than those required to inhibit the cimaterol response (yielding K_D_ values 126 and 37-fold higher respectively), again suggesting that cimaterol and CGP12177 agonist responses are occurring at different sites or conformations of the receptor (evidence (B) for SCE activation as outlined in the Introduction). Thus, this demonstrates the 2 pharmacologically observable conformation ensembles of the *β*1-AR: cimaterol is acting through the catecholamine conformation (the CCE) where agonist responses are readily inhibited by antagonists (including CGP12177), whereas the CGP12177 agonist response is occurring through a secondary conformation (an active-state SCE) where agonist responses are relatively resistant to inhibition. Finally, if increasing concentrations of CGP12177 are examined in the presence of a fixed concentration of cimaterol (Fig. 3E), it can be seen that low concentrations of CGP12177 inhibit the cimaterol response (due to high-affinity antagonism of the catecholamine conformation) whereas higher concentrations of CGP12177 stimulate an agonist response, as first shown by,^11^ evidence (D) for SCE activation as outlined in the Introduction. Bucindolol had a biphasic concentration response (Fig. 3F, evidence (C) for SCE activation as outlined in the Introduction).

The pharmacological response to other compounds fell into 4 distinct groups: **VL03/07**, **VL05/06/10**, **VL01/04/09,** and **VL08/11/12/13** (compound structures are shown in Fig. 1).

#### 4.3.2 Determination of the site of action of VL03 and VL07

**Fig. 4:**
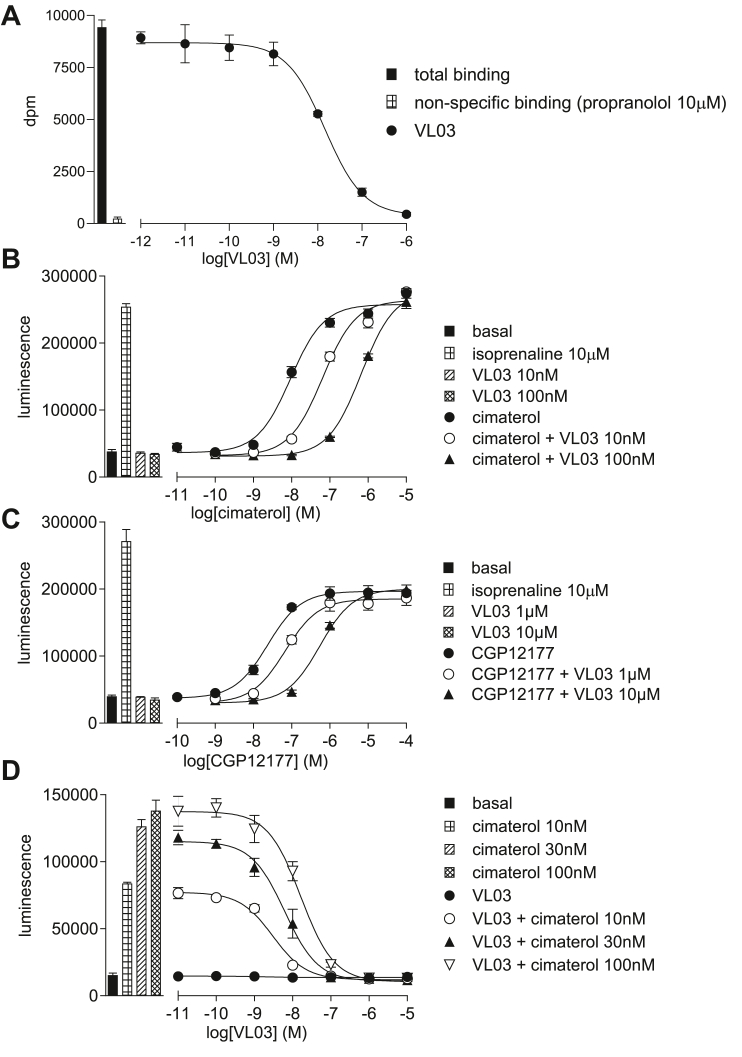
(A) Inhibition of ^3^H-CGP12177 whole-cell binding by **VL03** in CHO-*β*1-CRE-SPAP cells. The concentration of ^3^H-CGP12177 is 0.71 nM. (B–E) CRE-luciferase production in CHO-*β*1-CRE-luciferase cells in response to (B) cimaterol in the absence and presence of 10 nM and100 nM **VL03**; (C) CGP12177 in the absence and presence of 1 *μ*M and 10 *μ*M **VL03**; and (D) **VL03** in the absence and presence of 10, 30, or 100 nM cimaterol. Bars represent basal CRE-luciferase production and that in response to 10 *μ*M isoprenaline and other compounds alone as listed with each graph. Data points are mean ± SD of triplicate determinations and are representative of (A) 7, (B) 5, (C) 5, and (D) 5 separate experiments.

**VL03** and **VL07** inhibited cimaterol-induced responses in CHO-*β*1-CRE-luciferase cells with high affinity (Fig. 4B; Table 3). They both also inhibited the CGP12177-induced response but required higher concentrations to do so (Fig. 4C); thus again, these ligands had higher affinity for the catecholamine conformation ensemble (Table 3) of the *β*1-AR. **VL03** and **VL07** did not stimulate any agonist response themselves in CHO-*β*1-CRE-luciferase cells, although as expected from their affinity, low concentrations of **VL03** and **VL07** inhibited the cimaterol response as seen in Fig. 4D. There is therefore no evidence (A–D/Introduction) for SCE activation.

#### 4.3.3 Determination of the site of action of VL10, VL05, and VL06

**Fig. 5:**
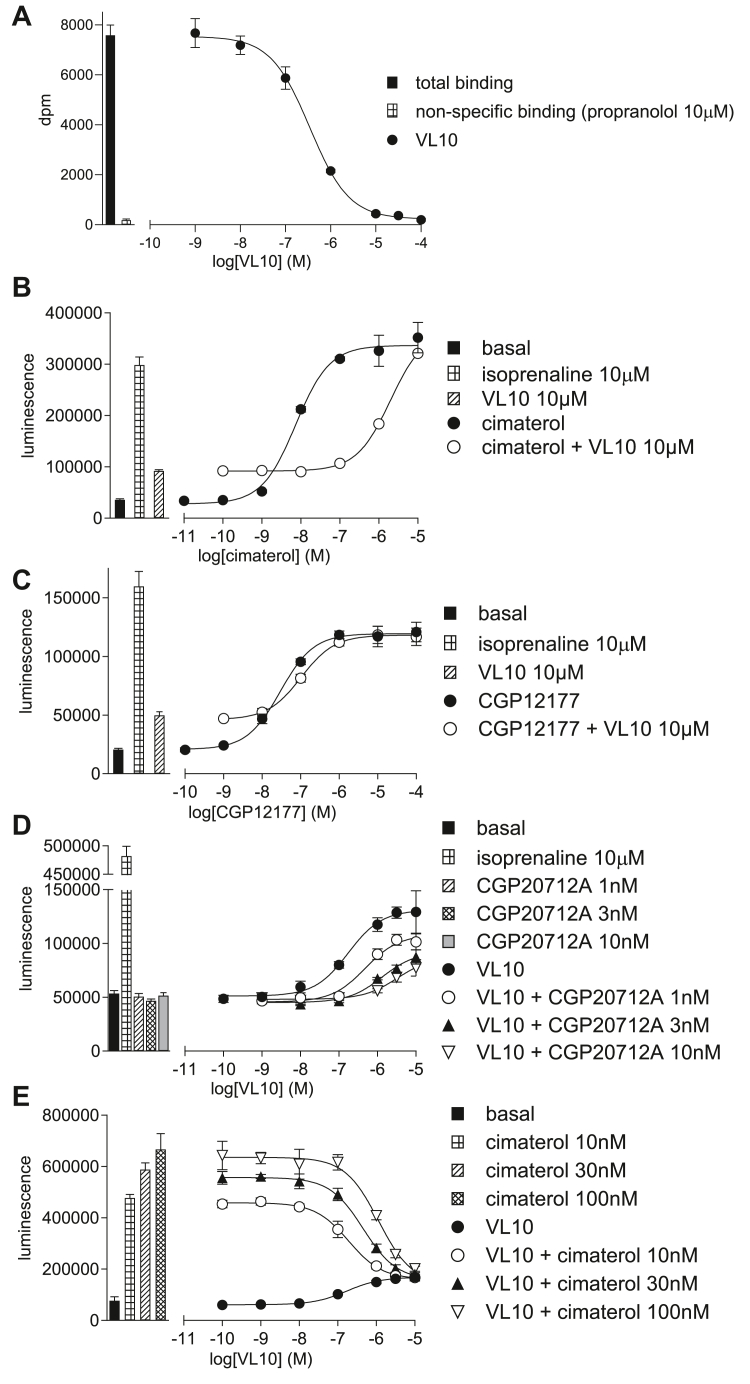
(A) Inhibition of ^3^H-CGP12177 whole-cell binding by **VL10** in CHO-*β*1-CRE-SPAP cells. The concentration of ^3^H-CGP12177 is 0.53 nM. (B–E) CRE-luciferase production in CHO-*β*1-CRE-luciferase cells in response to (B) cimaterol in the absence and presence of 10 *μ*M **VL10**; (C), CGP12177 in the absence and presence of 10 *μ*M **VL10**; (D) **VL10** in the absence and presence of 1, 3, or 10 nM CGP20712A; and (E) **VL10** in the absence and presence of 10, 30, or 100 nM cimaterol. Bars represent basal CRE-luciferase production and that in response to 10 *μ*M isoprenaline and other compounds alone as listed with each graph. Data points are mean ± SD of triplicate determinations and are representative of (A) 7, (B) 7, (C) 7, (D) 9, and (E) 7 separate experiments.

**VL10** (Fig. 5B), **VL05,** and **VL06** all inhibited cimaterol CHO-*β*1-CRE-luciferase responses, thus acting as antagonists of the catecholamine-stabilized conformation ensemble (Table 3). Even with the maximum concentration of ligand possible (10 *μ*M), this has little impact on the CGP12177 agonist response (Fig. 5C). Although the response to **VL06** was too small to accurately quantify, the agonist responses to **VL10** and **VL05** were readily antagonized by CGP20712A to yield K_D_ values of −9.73 and −9.54, respectively (Fig. 5D; Table 2). **VL10**, **VL05**, and **VL06** inhibited a fixed concentration of cimaterol at concentrations similar to those required to stimulate their agonist response. There is therefore no evidence (A–D) for SCE activation, and all the evidence suggests that *β*1-AR agonist interactions of **VL10**, **VL05,** and **VL06** are occurring through the same conformation as cimaterol, ie, the CCE.

#### 4.3.4 Determination of the site of action of VL01, VL04, and VL09

**Fig. 6:**
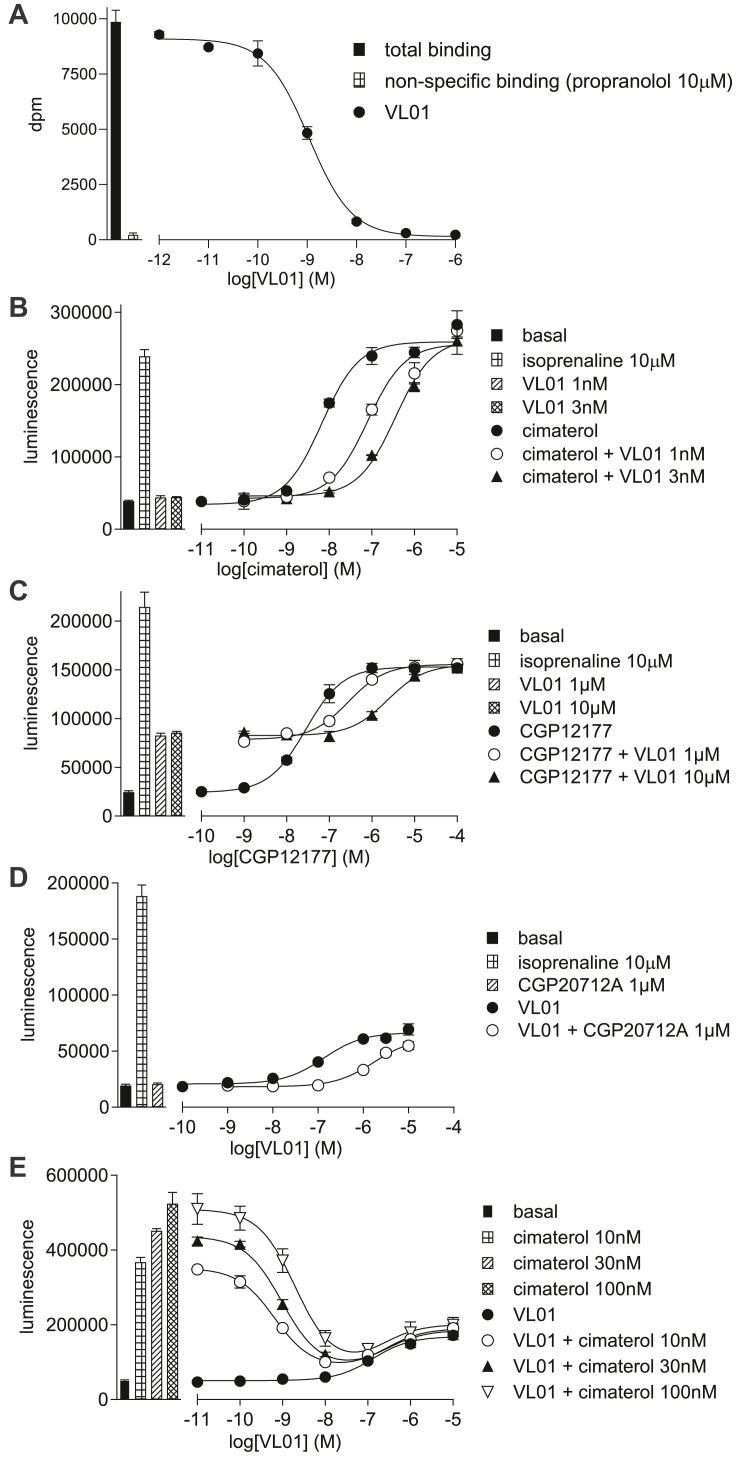
(A) Inhibition of ^3^H-CGP12177 whole-cell binding by **VL01** in CHO-*β*1-CRE-SPAP cells. The concentration of ^3^H-CGP12177 is 0.71 nM. (B–E) CRE-luciferase production in CHO-*β*1-CRE-luciferase cells in response to (B) cimaterol in the absence and presence of 1 and 3 nM **VL01**; (C) CGP12177 in the absence and presence of 1 and 10 *μ*M **VL01**; (D) **VL01** in the absence and presence of 1 *μ*M CGP20712A; and (E) **VL01** in the absence and presence of 10, 30, or 100 nM cimaterol. Bars represent basal CRE-luciferase production and that in response to 10 *μ*M isoprenaline and other compounds alone as listed with each graph. Data points are mean ± SD of triplicate determinations and are representative of (A) 7, (B) 6, (C) 5, (D) 9, and (E) 6 separate experiments.

**VL01** (Fig. 6B), **VL04,** and **VL09** inhibited cimaterol *β*1-CRE-luciferase responses with high affinity (log K_D_s −10.19, −9.12, and −8.84, respectively; Table 3). This is similar to the values obtained from ^3^H-CGP12177 whole-cell binding above, showing these compounds bind to the catecholamine-stabilized conformation ensemble with high affinity. **VL01** (Fig. 6C), **VL04** and **VL09** are also able to inhibit the CGP12177 *β*1-CRE-luciferase responses but with lower affinity (−6.64, −5.70, and −6.03, respectively) and are therefore able to bind to the SCE, but as with other antagonists, the affinity for this secondary conformation is less. From the high concentrations of **VL01**, **VL04,** and **VL09** required to inhibit the CGP12177 response (Fig. 6C), it is also evident that **VL01**, **VL04,** and **VL09** have agonist properties of their own.

**VL01** (Fig. 6D), **VL04,** and **VL09** all stimulated partial agonist responses in CHO-*β*1-luciferase cells (Table 2). Considerably higher concentrations of ligand were required to stimulate this response than inhibit the catecholamine conformation (evidence (A) for SCE activation). The concentration of antagonist (CGP20712A) required to inhibit these partial agonist responses was also high (Table 2; evidence (B) for SCE activation). When all 3 were examined in the presence of a fixed concentration of cimaterol, low concentrations inhibited the agonist actions of cimaterol, whereas higher concentrations were needed to stimulate the agonist response (Fig. 6E; evidence (D) for SCE activation). Thus **VL01**, **VL04,** and **VL09** were appearing pharmacologically similar to CGP12177, ie, having agonist actions via an SCE.

#### 4.3.5 Determination of the site of action of VL08, VL11, VL12, and VL13

**Fig. 7:**
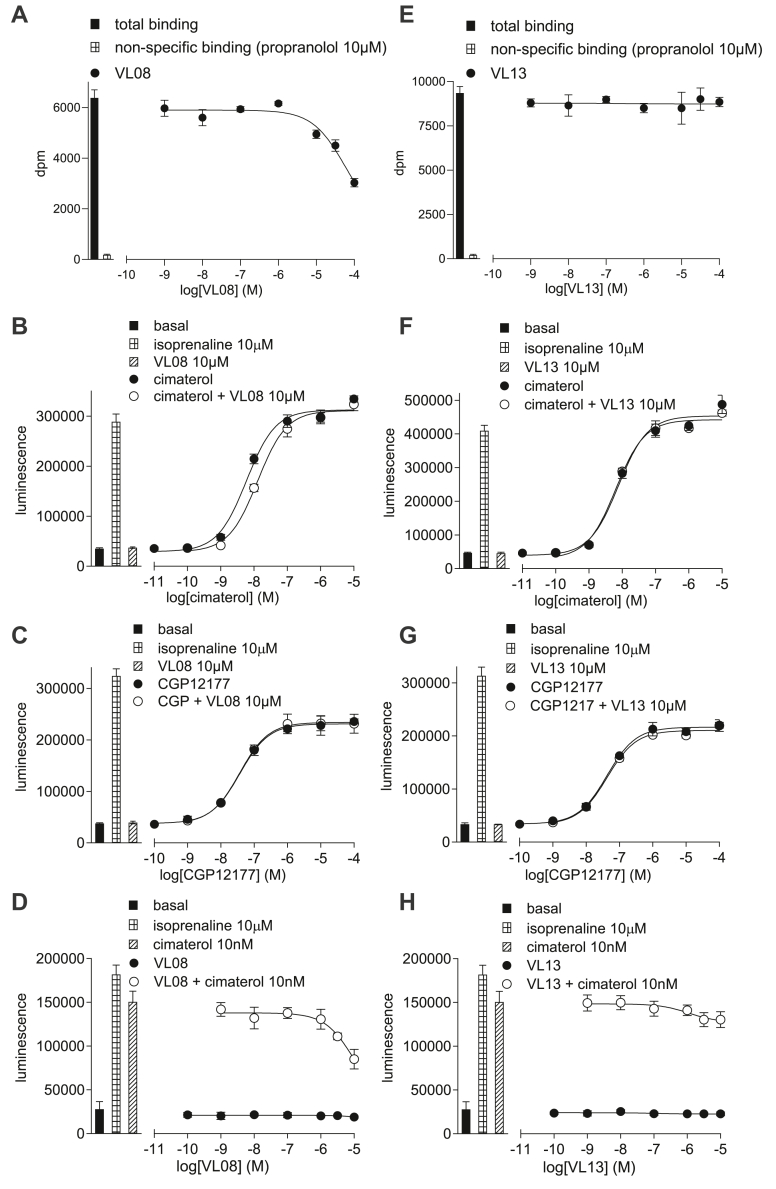
(A, E) Inhibition of ^3^H-CGP12177 whole-cell binding by (A) **VL08** and (E) **VL13** in CHO-*β*1-CRE-SPAP cells. The concentration of ^3^H-CGP12177 is (A) 0.62 nM and (E) 0.71 nM. (B–D, F–H) CRE-luciferase production in CHO-*β*1-CRE-luciferase cells in response to (B) cimaterol in the absence and presence of 10 *μ*M **VL08**; (C) CGP12177 in the absence and presence of 10 *μ*M **VL08**; (D) **VL08** in the absence and presence of 10 nM cimaterol; (F) cimaterol in the absence and presence of 10 *μ*M **VL13**; (C) CGP12177 in the absence and presence of 10 *μ*M **VL13**: (D) **VL13** in the absence and presence of 10 nM cimaterol. Bars represent basal CRE-luciferase production and that in response to 10 *μ*M isoprenaline and other compounds alone as listed with each graph. Data points are mean ± SD of triplicate determinations and are representative of (A) 5, (B) 5, (C) 5, (D) 5, (E) 5, (F) 6, (G) 5, and (H) 5 separate experiments.

The affinity of these compounds as determined by inhibition of ^3^H-CGP12177 was too low to be determined (Table 1). Although 10 *μ*M **VL08** and **VL11** was just about able to cause a rightward shift of the cimaterol response (Fig. 7), it was too small to quantify. **VL12** and **VL13** (10 *μ*M) were not able to inhibit the response at all (Fig. 7). None of the compounds were able to inhibit the CGP12177 response (up to 10 *μ*M, ie, the maximum concentration possible, Fig. 7). Likewise, they stimulated no agonist response, and minimal or no inhibition of the fixed cimaterol response was seen (Fig. 7). These ligands therefore barely interacted with the *β*1-AR at the CCE, and there is no evidence (A–D) of any SCE activation.

### 4.4 CHO-β1-CRE-SPAP and CHO-β2-CRE-SPAP responses

Ligands were evaluated in a separate second system (CRE-SPAP cells) for several reasons. First, this would confirm the *β*1-AR responses to ligands in a separate system, and show that the existence of the secondary site is not a product of receptor expression levels. Second, it allowed comparison between the *β*1-AR and *β*2-AR responses in the same cell background. Third, as a very different reporter product is being measured, it would demonstrate that the reporter protein itself is not interfering with the observed pharmacology. Finally, the CHO-*β*1-CRE-SPAP cell line has a high (supraphysiological) receptor expression level and so allows for the detection of small partial agonist responses, be it those occurring via the CCE or the SCE for the *β*1-AR. Responses in different cell lines with high and low receptor expression levels allow for further evidence (A–D) for SCE activation to be gathered.

Cimaterol stimulated a response in both CHO-*β*1-CRE-SPAP and CHO-*β*2-CRE-SPAP cells (Table 4) that was readily inhibited by CGP20712A in the CHO-*β*1 cells and by ICI118551 in the CHO-*β*2 cells, in keeping with previous studies, and thus demonstrating the presence of the respective receptors in each cell line (Table 5). In the CHO-*β*1-SPAP cells, the CGP12177 response (log EC_50_ −8.71 [Table 4] greater than log K_D_ −9.29 [Table 1], ie, evidence (A) for SCE activation) was (as expected with higher receptor expression levels) proportionately greater than in *β*1-luciferase cells (54% vs 74% isoprenaline) and left shifted (more potent; log EC_50_ −7.54 and −8.71) but once again required higher concentrations of antagonists to cause a rightward shift (yielding poorer K_D_ values, evidence (B) for SCE activation), demonstrating the presence of the SCE of the *β*1-AR. In the CHO-*β*2-cells, CGP12177 also stimulated a partial agonist CRE-SPAP response; however, this was readily inhibited by antagonists yielding similar log K_D_ values when measured as a shift of the cimaterol or CGP12177 response, demonstrating a lack of the CGP12177-induced SCE at the β2-AR (see ratios in Table 5). Alprenolol, oxprenolol, and pindolol, as previously reported,^19^^,^^24^ stimulated *β*1-biphasic responses (Table 4; evidence (C) for SCE activation).

**Table 4:** Agonist actions of compounds in CHO-*β*1-CRE-SPAP and CHO-*β*2-CRE-SPAP cells Log EC_50_ values are given, with % of the response compared with 10 *μ*M isoprenaline. Where obtained, the log K_D_ values for inhibition of agonist response by CGP20712 (*β*1) or ICI118551 (*β*2) are also given. The values are mean ± s.e.mean for n separate experiments.

	CHO-*β*1-CRE-SPAP					CHO-*β*2-CRE-SPAP Cells				
	Log EC_50_	% Isoprenaline	n	Log K_D_ CGP20712A	n	Log EC_50_	% Isoprenaline	n	Log K_D_ ICI118551	n
Alprenolol^a^	EC_50_1 *−*8.34 ± 0.07 EC_50_2 *−*6.44 ± 0.14 62.5 ± 2.7% at EC_50_1	56.7 ± 3.7	8	*−*8.44 ± 0.11	7	*−*9.58 ± 0.06	49.9 ± 2.2	6	*−*9.68 ± 0.03	5
Bucindolol	*−*9.12 ± 0.04	84.5 ± 2.4	9	*−*7.68 ± 0.09	9	*−*9.70 ± 0.04	70.3 ± 2.0	8	*−*8.81 ± 0.03	6
CGP12177	*−*8.71 ± 0.08	74.0 ± 3.7	17	*−*7.02 ± 0.12	12	*−*9.57 ± 0.08	53.0 ± 2.4	19	*−*9.56 ± 0.06	10
Cimaterol	*−*9.26 ± 0.05	82.8 ± 3.1	20	*−*8.96 ± 0.12	17	*−*10.14 ± 0.05	95.3 ± 2.1	16	*−*9.76 ± 0.03	11
Oxprenolol^a^	EC_50_1 *−*8.41 ± 0.03 EC_50_2 *−*5.68 ± 0.11 79.8 ± 2.4% at EC_50_1	67.8 ± 3.9	7	*−*8.77 ± 0.07	7	*−*9.42 ± 0.05	40.1 ± 2.3	8	*−*9.92 ± 0.08	6
Pindolol^a^	EC_50_1 *−*8.75 ± 0.05 EC_50_2 *−*5.91 ± 0.11 62.7 ± 2.4% at EC_50_1	81.0 ± 2.6	11	*−*8.59 ± 0.11	7	*−*9.73 ± 0.07	61.8 ± 2.8	7	*−*9.87 ± 0.08	6
**VL01**	*−*8.56 ± 0.10	69.6 ± 2.5	14	*−*7.59 ± 0.09	20	*−*9.85 ± 0.08	52.1 ± 3.5	14	*−*9.13 ± 0.05	23
**VL03**	No response		5			No response		5		
**VL04**^a^	EC_50_1 *−*9.06 ± 0.13 EC_50_2 *−*6.39 ± 0.14 52.1 ± 2.8% at EC_50_1	75.2 ± 3.6	18	*−*8.59 ± 0.11	12	*−*9.59 ± 0.13	55.1 ± 2.7	11	*−*9.53 ± 0.04	13
**VL05**	*−*8.30 ± 0.06	62.3 ± 3.8	13	*−*9.06 ± 0.15	12	*−*8.42 ± 0.09	63.2 ± 3.2	13	*−*9.78 ± 0.10	17
**VL06**	*−*6.47 ± 0.10	53.6 ± 3.1	15	*−*9.11 ± 0.08	8	*−*6.44 ± 0.15	15.2 ± 1.2	12		
**VL07**	*−*6.55 ± 0.17	26.5 ± 1.9	12			*−*9.30 ± 0.14	13.1 ± 2.0	10		
**VL08**	No response		5			No response		6		
**VL09**^a^	EC_50_1 *−*9.15 ± 0.08 EC_50_2 *−*6.61 ± 0.21 69.4 ± 3.2% at EC_50_1	80.1 ± 4.1	13	*−*8.50 ± 0.11	12	*−*9.35 ± 0.13	65.8 ± 4.0	11	*−*9.84 ± 0.07	14
**VL10**	*−*7.73 ± 0.07	66.9 ± 2.5	19	*−*8.88 ± 0.12	15	*−*7.40 ± 0.06	28.7 ± 2.0	13	*−*9.93 ± 0.09	9
**VL11**	No response		5			No response		6		
**VL12**	No response		5			No response		5		
**VL13**	No response		6			No response		4		

a Alprenolol, oxprenolol, pindolol, **VL04,** and **VL09** stimulated biphasic concentration-response curves in the CHO-*β*1-CRE-SPAP cells (Fig. 11). The log EC_50_ at both components are given, along with % occurring via EC_50_1 and % of isoprenaline of the whole response. The K_D_ value for CGP20712A is for inhibition of the first component.

**Table 5:** Log K_D_ values of ligands determined from a rightward shift of agonist responses to cimaterol or CGP12177 in CHO-*β*1-CRE-SPAP and CHO-*β*2-CRE-SPAP cells Values are calculated using the method of Gaddum (eq. 5). The values are mean ± s.e.mean for n separate experiments. The ratio of affinities for the 2 conformations is also shown (as in Table 3). Whereas this ratio shows that ligand affinity for the 2 conformations varies between 65 and 316-fold in the CHO-*β*1 cells, the differences in antagonist affinity when cimaterol and CGP12177 are agonists in the CHO-*β*2 cells are only 1.6 to 3.8-fold in keeping with a single conformation.

	CHO-*β*1-CRE-SPAP					CHO-*β*2-CRE-SPAP				
	Log K_D_ Catecholamine conformation Cimaterol as Agonist	n	Log K_D_ Secondary Conformation CGP12177 as Agonist	n	Log Ratio of Affinities	Log K_D_ (Cimaterol as Agonist)	n	Log K_D_ (CGP12177 as Agonist)	n	Log Ratio of Affinities
CGP20712A	*−*8.96 ± 0.12	17	*−*7.02 ± 0.12	12	1.94	*−*6.05 ± 0.08	6	*−*5.74 ± 0.14	6	0.31
ICI118551	*−*7.30 ± 0.07	10	*−*5.49 ± 0.10	6	1.81	*−*9.76 ± 0.03	11	*−*9.56 ± 0.06	10	0.20
**VL01**	*−*10.17 ± 0.14^a^	14	b			*−*11.08 ± 0.13^a^	16	b		
**VL03**	*−*8.75 ± 0.05	11	*−*6.25 ± 0.06	10	2.50	*−*10.33 ± 0.04	21	*−*10.12 ± 0.05	30	0.21
**VL04**	*−*9.15 ± 0.11^a^	13	b			*−*9.94 ± 0.07^a^	16	b		
**VL05**	b		b			b		b		
**VL06**	b		b			*−*6.34 ± 0.17^a^	8	*−*5.93 ± 0.15	4	0.41
**VL07**	*−*8.59 ± 0.05	17	*−*6.19 ± 0.11	8	2.40	*−*9.47 ± 0.06	17	*−*8.89 ± 0.07	14	0.58
**VL08**	No shift 10 *μ*M	5	No shift 10 *μ*M	5		*−*5.68 ± 0.14	7	No shift 10 *μ*M	5	
**VL09**	*−*9.71 ± 0.20^a^	12	b			*−*9.36 ± 0.10^a^	12	b		
**VL10**	*−*6.93 ± 0.25	7	b			*−*7.43 ± 0.09	7	*−*6.86 ± 0.10	10	0.57
**VL11**	No shift 10 *μ*M	6	No shift 10 *μ*M	6		No shift 10 *μ*M	5	No shift 10 *μ*M	5	
**VL12**	No shift 10 *μ*M	5	No shift 10 *μ*M	5		*−*5.43 ± 0.26	5	No shift 10 *μ*M	5	
**VL13**	No shift 10 *μ*M	4	No shift 10 *μ*M	5		No shift 10 *μ*M	5	No shift 10 *μ*M	4	

a where the ligand had partial agonism and therefore the K_D_ was calculated using the method of Stephenson (eq. 7).

b The partial agonist stimulation was too great to enable a calculation of K_D_ value.

### 4.5 VL03 and VL07

**Fig. 8:**
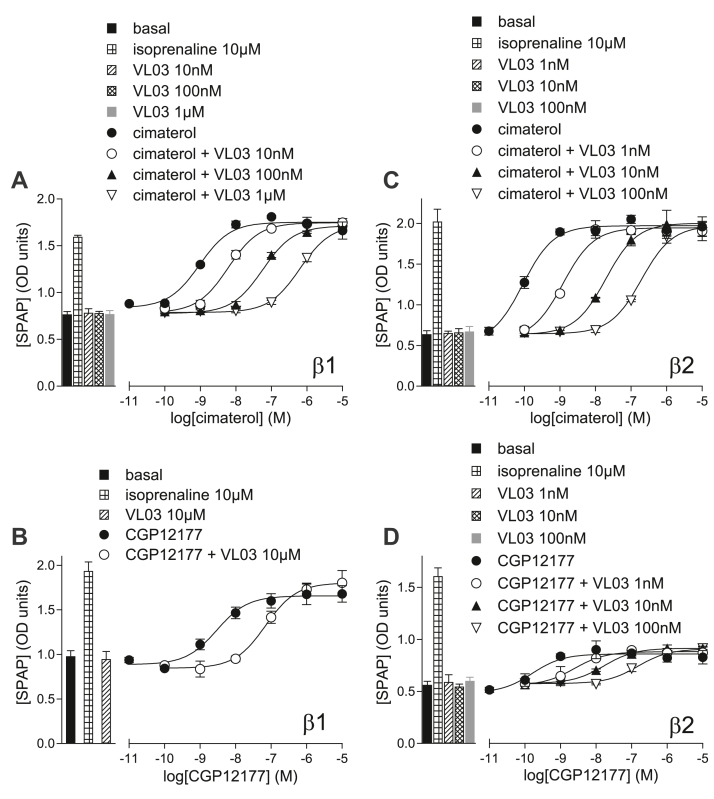
CRE-SPAP production in (A) and (B) CHO-*β*1-CRE-SPAP cells and (C) and (D) CHO-*β*2-CRE-SPAP cells in response to (A) and (C) cimaterol in the absence and presence of 1 nM, 10 nM, 100 nM, and 1 *μ*M **VL03**, and (B) and (D) CGP12177 in the absence and presence of 1 nM, 10 nM, 100 nM, and 10 *μ*M **VL03**. Bars represent basal CRE-SPAP production and that in response to 10 *μ*M isoprenaline and other compounds alone as listed with each graph. Data points are mean ± SD of triplicate determinations and are representative of (A) 7, (B) 8, (C) 7, and (D) 10 separate experiments. The Schild slopes were (A) 0.96 ± 0.05 (*n* = 7), (C) 0.99 ± 0.04 (*n* = 7), and (D) 1.00 ± 0.03 (*n* = 7).

**VL03** did not stimulate an agonist response in either cell line (Table 4); however, it inhibited the cimaterol responses in both CHO-*β*1 and CHO-*β*2 cells with high affinity, as expected from its inhibition of ^3^H-CGP12177 (Fig. 8; Tables 1 and 5). **VL03** also inhibited the CGP12177 agonist responses. The *β*2-response was readily inhibited to yield a similar K_D_ value for ICI118551 very similar to when cimaterol was the agonist, suggesting both cimaterol and CGP12177 are acting through the same conformation, whereas the CHO-*β*1 CGP12177 response required a higher concentration of **VL03** to cause a rightward shift, yielding a poorer K_D_ value, in keeping with lower affinity inhibition of the SCE as for most *β*-antagonists (Fig. 8; Table 5). There is therefore no evidence (A–D) for any SCE activation. **VL07** stimulated small agonist responses in both cell lines (Table 4) as previously reported,^19^ and comparison of its EC_50_ and K_D_ would suggest that its *β*1-AR agonist response is occurring via an SCE (evidence (A) for SCE activation).

### 4.6 VL10, VL05, and VL06

**Fig. 9:**
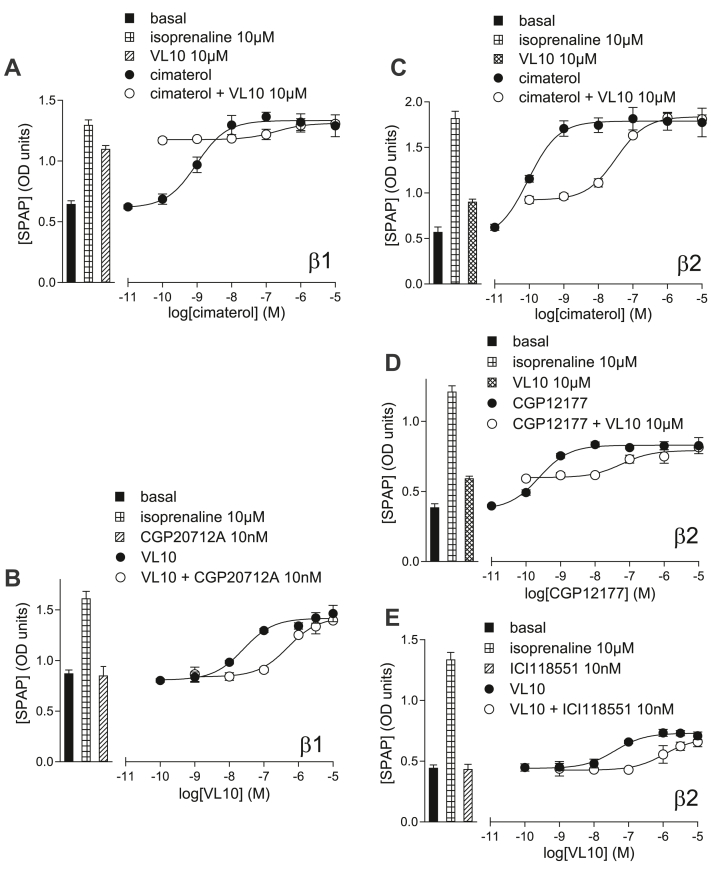
CRE-SPAP production in (A) and (B) CHO-*β*1-CRE-SPAP cells and (C–E) CHO-*β*2-CRE-SPAP cells in response to (A) and (C) cimaterol in the absence and presence of 10 *μ*M **VL10** and (D) CGP12177 in the absence and presence of 10 *μ*M **VL10** and (B) and (E) **VL10** in the absence and presence (B) 10 nM CGP20712A and (E) 10 nM ICI118551. Bars represent basal CRE-SPAP production and that in response to 10 *μ*M isoprenaline and other compounds alone as listed with each graph. Data points are mean ± SD of triplicate determinations and are representative of (A) 7, (B) 13, (C) 7, (D) 10, and (E) 8 separate experiments.

As expected, the partial agonist response to **VL10** was one again substantially greater in the high expressing *β*1-SPAP cells (67% isoprenaline maximum vs 14% in the CHO-*β*1-CRE-luciferase cells; Fig. 9; Table 4), but K_D_ values for inhibition of cimaterol and CGP12177 were just possible to determine. The agonist response to **VL10** response was however readily inhibited by CGP20712A. The agonist responses to **VL05** and **VL06** were likewise proportionally greater, and therefore allowing inhibition by CGP20712A to be evaluated. **VL10**, **VL05,** and **VL06** were all readily inhibited by CGP20712A, suggesting that these agonist responses were occurring via the CCE with no evidence (A–D) for any SCE activation.

### 4.7 VL01, VL04, and VL09

**Fig. 10:**
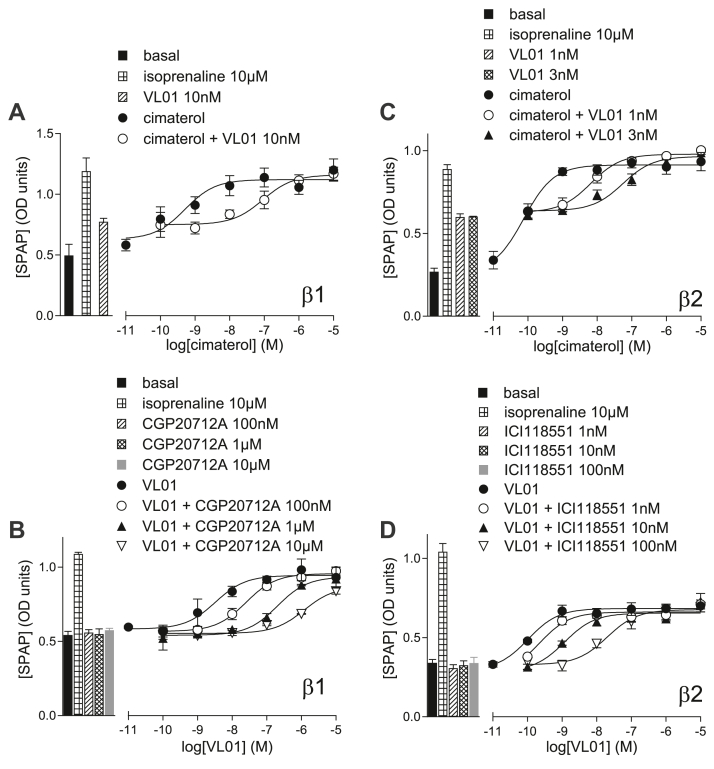
CRE-SPAP production in (A, B) CHO-*β*1-CRE-SPAP cells and (C, D) CHO-*β*2-CRE-SPAP cells in response to (A, C) cimaterol in the absence and presence of 1, 3, and 10 nM **VL01**, and (B, D) **VL01** in the absence and presence of (B) 100 nM, 1 *μ*M, and 10 *μ*M CGP20712A and (D) 1, 10, and 100 nM ICI118551. Bars represent basal CRE-SPAP production and that in response to 10 *μ*M isoprenaline and other compounds alone as listed with each graph. Data points are mean ± SD of triplicate determinations and are representative of (A) 10, (B) 11, (C) 9, and (D) 11 separate experiments. The Schild slopes were (B) 0.94 ± 0.17 (*n* = 4) and (D) 0.96 ± 0.07 (*n* = 4).

**VL01** was able to inhibit the *β*1-cimaterol response to yield a K_D_ value similar to that measured by ^3^H-CGP12177 whole-cell binding (Fig. 10), suggesting high-affinity binding to the catecholamine conformation. However, its log EC_50_ − 8.56 (Table 4) was substantially higher than its K_D_ measurements (log K_D_ − 9.45 [Table 1] and log K_D_ − 10.17 [Table 5], evidence (A) for SCE activation). Its partial agonism was similar to that of CGP12177, rendering a measurement of affinity at the SCE unobtainable. In the CHO-*β*1-CRE-SPAP cells, **VL01** responses were more resistant to inhibition (evidence (B) for SCE activation), yielding K_D_ values for CGP20712A similar to those when CGP12177 was the agonist (Fig. 10; Tables 4 and 5). In the CHO-*β*2-CRE-SPAP cells, **VL01**-stimulated *β*2-agonist responses were readily inhibited by ICI118551 to yield a similar value to those obtained when cimaterol and CGP12177 were agonists. This suggests that all ligands are acting at the same *β*2-conformation (Fig. 10; Tables 4 and 5).

**Fig. 11:**
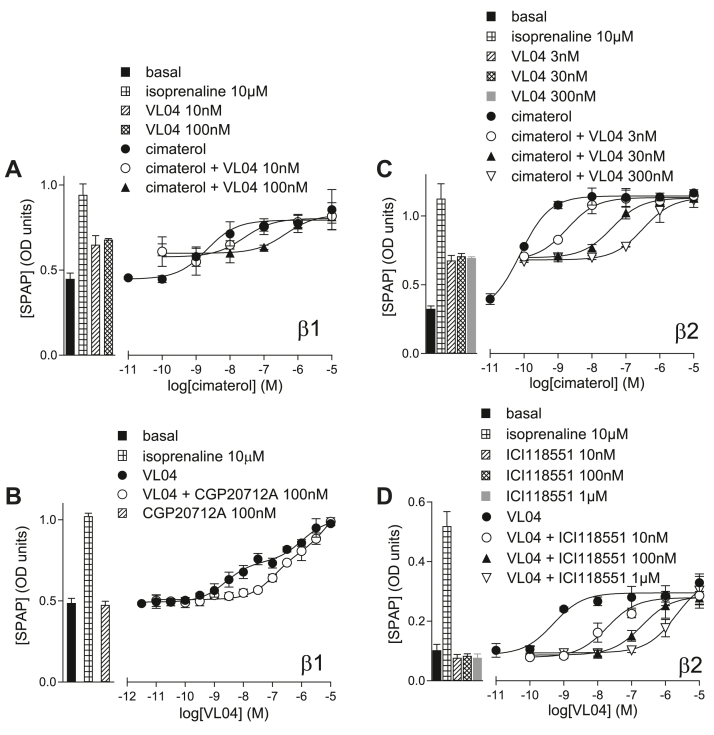
CRE-SPAP production in (A, B) CHO-*β*1-CRE-SPAP cells and (C, D) CHO-*β*2-CRE-SPAP cells in response to (A) and (C) cimaterol in the absence and presence of 3, 10, 30, 100, and 300 nM **VL04**, and (B, D) **VL04** in the absence and presence of (B) 100 nM CGP20712A and (D) 10 nM, 100 nM, and 1 *μ*M ICI118551. Bars represent basal CRE-SPAP production and that in response to 10 *μ*M isoprenaline and other compounds alone as listed with each graph. Data points are mean ± SD of triplicate determinations and are representative of (A) 7, (B) 11, (C) 6, and (D) 6 separate experiments. The Schild slope for (D) was 1.11 ± 0.07 (*n* = 3).

**VL04** and **VL09** inhibited cimaterol responses in both the *β*1 and *β*2 cell lines, but again their increased partial agonist response meant that measurements of **VL04** and **VL09** antagonist affinity in the presence of CGP12177 were not possible. In the CHO-*β*2 cells, **VL04** and **VL09** agonist responses were readily inhibited by ICI118551, yielding similar K_D_ values to all others obtained, suggesting all compounds were interacting at the same conformation. In the CHO-*β*1-CRE-SPAP cells, both of these compounds had a biphasic concentration-response curve (Fig. 11, evidence (C) for SCE activation). The first component of this was more readily inhibited by CGP20712A than the secondary component, in a manner very similar to that seen with pindolol.^19^

### 4.8 β1-WT and β1V189T-L195Q-W199Y mutation data

A previous study has suggested that certain amino acids located at the extracellular end of TM4 (V189, L195, and W199) are important for the *β*1-SCE interaction of CGP12177 and pindolol.^25^ These are the same residues that also emerged from the unbiased docking calculations reported above. When these were mutated to those of the *β*2-AR (namely T, Q, and Y respectively), CGP12177 was no longer able to activate secondary site response and appeared to interact with the *β*1-AR as a conventional partial agonist at the same site as cimaterol. The biphasic partial agonist responses to pindolol also became monophasic suggesting that binding to the secondary site was no longer possible. Thus, in the *β*1-V189T-L195Q-W199Y receptor, all evidence (A–D) for SCE activation was lost.^25^ **VL01**, **VL04,** and **VL09** responses were therefore compared in stable mixed populations of cells expressing the *β*1-WT or the triple mutant *β*1-V189T-L195Q-W199Y receptor.

### 4.9 ^3^H-CGP12177 whole-cell binding in β1-WT and β1-V189T-L195Q-W199Y cells

**Fig. 12:**
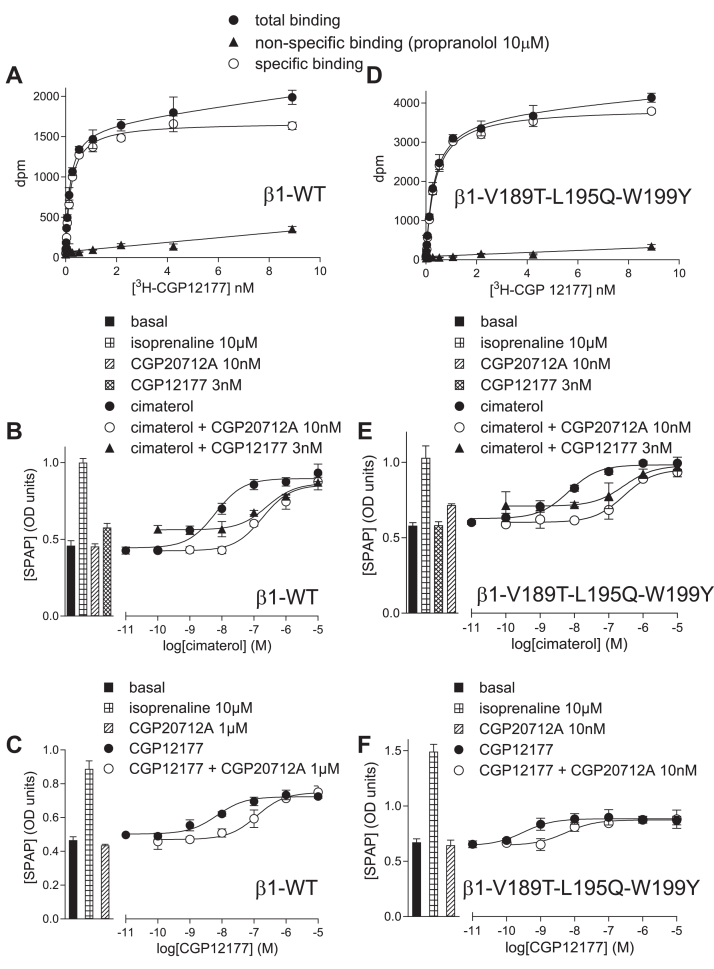
(A) and (D) ^3^H-CGP12177 saturation binding in stable mixed population of CHO cells expressing (A) *β*1-AR and (D) *β*1-V189T-L195Q-W199Y mutant receptor. (B, C, E, and F) CRE-SPAP production in stable mixed population of CHO cells expressing (B, C) *β*1-AR and (E, F) *β*1-V189T-L195Q-W199Y mutant receptor, (B) and (E) in response to cimaterol in the absence and presence 10 nM CGP20712A and 3 nM CGP1277 and (C) and (F) CGP12177 in the absence and presence of (C) 1 *μ*M CGP210712A and (F) 10 nM CGP20712A. Bars represent basal CRE-SPAP production and that in response to 10 *μ*M isoprenaline and other compounds alone as listed with each graph. Data points are mean ± SD of triplicate determinations and are representative of (A) 6, (B) 6, (C) 9, (D) 6, (E) 6, and (F) 9 separate experiments.

^3^H-CGP12177 saturation binding in 6 stable mixed populations expressing the *β*1-AR and 6 expressing the *β*1-V189T-L195Q-W199Y receptors yielded K_D_ values for ^3^H-CGP12177 of 0.17 ± 0.01 nM (*n* = 6) and 0.27 ± 0.02 nM (*n* = 6), respectively (Fig. 12). From competition binding, the K_D_ values obtained for cimaterol, CGP20712A, **VL01**, **VL04,** and **VL09** were similar with both receptors (Table 6). However, as expected, ICI118551 had higher affinity for the *β*1-V189T-L195Q-W199Y mutant than *β*1-WT (Table 6). As previously noted, the presence of the V189T mutation (either alone or in combination with other mutations)^25^ increases the affinity of ICI118551, thus distinguishing these populations of cells from those expressing the *β*1-WT receptor.

**Table 6:** Affinity of *β*-adrenoceptor ligands for the *β*1-WT and *β*1-V189T-L195Q-W199Y receptors expressed in stable mixed populations of CHO cells obtained from ^3^H-CGP12177 whole-cell binding (left half) and from rightward shift of a cimaterol CRE-SPAP response (right half) Values are mean ± s.e.mean for n separate experiments. From binding studies, for affinity of ^3^H-CGP12177 (K_D_) was determined from saturation binding and was 0.170 ± 0.007 nM (log −9.77, *n* = 6) for *β*1-WT and 0.272 ± 0.020 nM (log −9.57, *n* = 6) for *β*1-V189T-L195Q-W199Y. A higher affinity for ICI118551 in *β*1-V189T-L195Q-W199Y compared with *β*1-WT was expected as all V189T mutant receptors have this property.^25^

	^3^H-CGP12177 whole cell binding *β*1-WT		^3^H-CGP12177 whole cell binding *β*1-V189T-L195Q-W199Y		CRE-SPAP production *β*1-WT		CRE-SPAP production *β*1-V189T-L195Q-W199Y	
	Log K_D_	n	Log K_D_	n	Log K_D_	n	Log K_D_	n
CGP12177	(*−*9.77)		(*−*9.57)		*−*10.11 ± 0.07^a^	6	*−*10.28 ± 0.11^a^	6
CGP20712A	*−*9.40 ± 0.03	6	*−*9.50 ± 0.06	6	*−*9.44 ± 0.07	6	*−*9.80 ± 0.12	6
Cimaterol	*−*6.63 ± 0.06	7	*−*6.76 ± 0.05	7				
ICI118551	*−*6.95 ± 0.07	7	*−*7.60 ± 0.02	7	*−*7.04 ± 0.11	6	*−*8.14 ± 0.12	6
**VL01**	*−*9.85 ± 0.03	8	*−*9.56 ± 0.03	8	*−*10.24 ± 0.10^a^	9	*−*10.09 ± 0.06^a^	10
**VL04**	*−*8.93 ± 0.03	10	*−*8.76 ± 0.03	10	*−*9.30 ± 0.09^a^	10	*−*9.56 ± 0.06^a^	11
**VL09**	*−*8.70 ± 0.04	9	*−*8.61 ± 0.02	9	*−*9.17 ± 0.11^a^	10	*−*9.27 ± 0.07^a^	11

a where the ligand had partial agonism and therefore the K_D_ was calculated using the method of Stephenson (eq. 7).

### 4.10 CRE-SPAP responses in β1-WT and β1-V189T-L195Q-W199Y cells

At both receptors, the cimaterol responses were inhibited by both CGP20712A and CGP12177 with high affinity, yielding log K_D_ value similar to those obtained from ^3^H-CGP12177 whole-cell binding (Fig. 12; Tables 6 and 7) and similar to those obtained in the stable cell lines. As expected, the affinity of ICI118551 was increased in the triple mutant compared with *β*1-WT. In the *β*1-WT cells, the CGP12177 response again required higher concentrations of antagonist to inhibit the response (evidence (B) for SCE activation), and the CGP12177 response (log EC_50_ −8.30) was itself right-shifted compared with the K_D_ (log K_D_ − 9.77; evidence (A) for SCE activation), again confirming the presence of the SCE in these stable mixed populations of cells. In the *β*1-V189T-L195Q-W199Y cells, however, the partial agonist CGP12177 response (log EC_50_ –9.67) was similar to the K_D_ value in these cells (log K_D_ − 9.57 measured from ^3^H-CGP12177 binding above = loss of evidence (A) compared with *β*1-WT). Furthermore, the CGP12177 response is more readily inhibited by CGP20712A, yielding K_D_ values for CGP20712A similar to that obtained when cimaterol was the agonist (= loss of evidence (B) compared with *β*1-WT; Fig. 12; Table 7). Overall, this suggests that at the *β*1-V189T-L195Q-W199Y receptor, CGP12177 is stimulating its agonist response via the same conformation as cimaterol (as seen in the *β*2-AR). Thus, in the *β*1-V189T-L195Q-W199Y receptor, the SCE appears absent, while leaving the CCE pharmacologically intact.

**Table 7:** Agonist actions of compounds obtained from *β*1-WT and *β*1-V189T-L195Q-W199Y receptors expressed in stable mixed populations of CHO cells Log EC_50_ values are given, with % of the response compared to 10 *μ*M isoprenaline. Log K_D_ values for inhibition of the agonist response by CGP20712A are also given. The values are mean ± s.e.mean for n separate experiments.

	*β*1-WT					*β*1-V189T-L195Q-W199Y				
	Log EC_50_	% isoprenaline	n	Log K_D_ CGP20712A	n	Log EC_50_	% isoprenaline	n	Log K_D_ CGP20712A	n
Cimaterol	*−*8.25 ± 0.04	82.8 ± 1.5	8	*−*9.44 ± 0.07	6	*−*8.33 ± 0.05	79.2 ± 2.1	9	*−*9.80 ± 0.12	6
CGP21177	*−*8.30 ± 0.06	45.2 ± 2.8	9	*−*7.37 ± 0.09	13	*−*9.67 ± 0.06	27.9 ± 1.7	9	*−*9.30 ± 0.10	13
										
**VL01**^a^	EC_50_1 *−*9.65 ± 0.11 Log EC_50_2 *−*6.70 ± 0.12 49.0 ± 2.3 % at EC_50_1	42.9 ± 3.0	15	*−*8.42 ± 0.14	17	*−*9.59 ± 0.06	30.4 ± 1.3	16	*−*8.84 ± 0.13	20
**VL04**^a^	EC_50_1 *−*9.10 ± 0.14 EC_50_2 *−*6.22 ± 0.14 41.9 ± 2.6 % at EC_50_1	39.5 ± 3.0	13	*−*8.79 ± 0.18	11	*−*9.18 ± 0.09	26.7 ± 1.6	16	*−*9.18 ± 0.14	12
**VL09**^a^	EC_50_1 *−*9.29 ± 0.15 EC_50_2 *−*6.76 ± 0.14 53.9 ± 3.3% at EC_50_1	45.2 ± 4.7	10	*−*8.79 ± 0.13	11	*−*9.20 ± 0.09	35.6 ± 1.8	9	*−*9.43 ± 0.16	11

a **VL01**, **VL04,** and **VL09** stimulated a biphasic concentration-response curve in the *β*-WT cells (Fig. 13). The log EC_50_ at both components are given, along with along with % occurring via EC_50_1 and % of isoprenaline of the whole response. The K_D_ value for CGP20712A is for inhibition of the first component.

When the responses to **VL04** and **VL09** were examined in these cells (Fig. 13; Table 7), a biphasic response was seen for both ligands in the *β*1-WT stable mixed populations of cells, with the first component being more readily inhibited than the second, just as in the stable CHO-*β*1-CRE-SPAP cell line (evidence (C) for SCE activation). In the *β*1-V189T-L195Q-W199Y cells, the response was a monophasic partial agonist response (= loss of evidence (C) compared with *β*1-WT) that was readily inhibited by CGP20712A; thus, the more antagonist-resistant secondary component appeared to be missing (Fig. 13; Table 7, = loss of evidence (B) for SCE activation). For **VL01**, in contrast to the response in the stable cell lines, the response in the *β*1-WT stable mixed population appeared biphasic (evidence (C) for SCE activation), although the antagonist-resistant secondary component also appeared to have disappeared in the *β*1-V189T-L195Q-W199Y cells (= loss of evidence (C) compared with *β*1-WT.

**Fig. 13:**
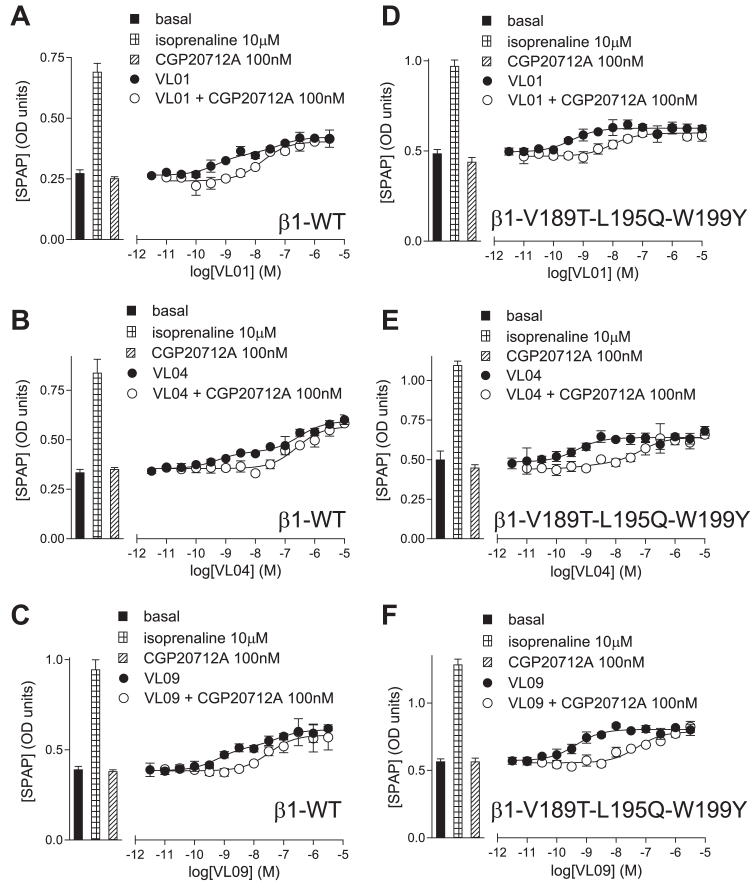
CRE-SPAP production in stable mixed population of CHO cells expressing (A–C) *β*1-AR and (D–F) *β*1-V189T-L195Q-W199Y mutant receptor in response to (A) and (D) **VL01** in the absence and presence 100 nM CGP20712A, (B) and (E) **VL04** in the absence and presence 100 nM CGP210712A and (C) and (F) **VL09** in the absence and presence of 100 nM CGP20712A. Bars represent basal CRE-SPAP production and that in response to 10 *μ*M isoprenaline and other compounds alone as listed with each graph. Data points are mean ± SD of triplicate determinations and are representative of (A) 12, (B) 11, (C) 11, (D) 12, (E) 12, and (F) 11 separate experiments.

### 4.11 Lack of responses in CHO-CRE-SPAP cells

In CHO-CRE-SPAP cells (ie, cells expressing the reporter gene but no transfected receptor), whereas forskolin stimulated a response that was 3.0 ± 0.1-fold over basal (*n* = 5), there were no responses to any of the VL compounds (*n* = 5 each ligand). As previously reported, there are no responses to cimaterol, CGP12177, ICI118551, or CGP20712A in these cells, either. This confirms that all responses seen were occurring via the transfected receptors.

## 5 Discussion

The *β*2-AR appears as a conventional receptor. CGP12177 affinity (^3^H-CGP12177 binding log K_D_ −9.77 from saturation and −9.75 from competition binding) is similar to its partial agonist log EC_50_ value (−9.57). Responses are similarly inhibited (ICI118551 log K_D_ of −9.76 when cimaterol, and −9.56 when CGP12177 is the agonist) suggesting interaction at a single receptor conformation. **VL03** was a high-affinity neutral antagonist. **VL07**, **VL06**, **VL10**, **VL04**, **VL01**, **VL09,** and **VL05** had increasing degrees of partial agonism (with responses readily inhibited by ICI118551), whereas **VL08**, **VL11**, **VL12**, and **VL13** barely interacted with the receptor (all compounds depicted in Fig. 1). Thus, VL compounds behaved as conventional *β*2-ligands interacting at a single conformation—the CCE/IBS.

For *β*1-ARs, several pharmacological anomalies reveal an agonist-stabilized SCE. First, CGP12177’s high affinity (eg, log K_D_ −9.87 from inhibition of cimaterol in *β*1-CRE-luciferase cells) differs from its partial agonist log EC_50_ value (−7.54), suggesting CGP12177 agonist activation via a different conformation (an SCE, evidence (A)). Second, CGP12177 agonist responses are relatively resistant to inhibition (required 126-fold higher CGP20712A concentrations to inhibit CGP12177 than cimaterol responses), suggesting CGP12177 stimulation is *not* via the CCE (evidence (B)). Third, low CGP12177 concentrations inhibit cimaterol but high CGP21177 concentrations stimulate a response (Fig. 3E, evidence (D)), which cannot be reconciled with single conformation interaction. Thus, 2 *β*1-AR conformations are demonstrated: a high-affinity CCE where agonist responses are readily antagonized, and a low-affinity CGP12177-stabilized SCE, where agonist responses are more resistant to antagonism.^1^

**Fig. 14:**
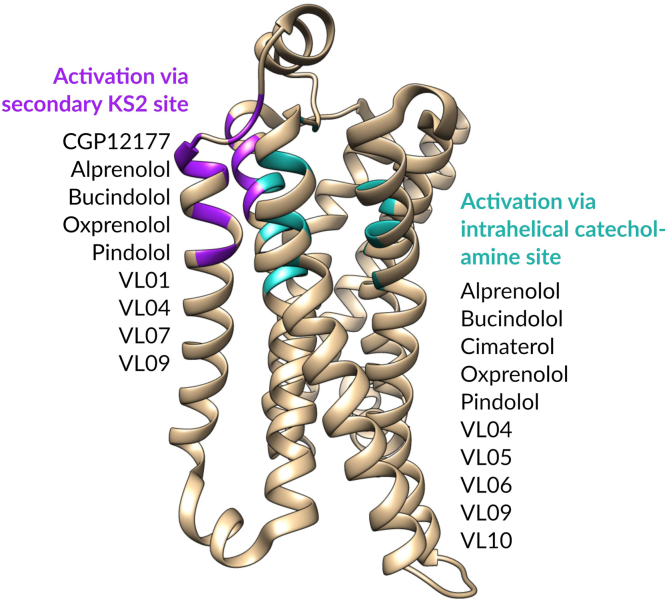
Summary of the findings: the protein shown is PDB ID 7BVQ, and the IBS has been colored in cyan, and was defined as any residue within 5 Å around the ligand in the structure, carazolol. Residues in magenta are the ones that emerged from our docking calculations, and correspond to KS2. Compound classification is according to the pharmacological results reported in this work.

Although several ligands stimulate *β*1-AR SCE responses, the structure-activity relationship (SAR) surrounding SCE interaction is relatively unknown. CGP12177 (most studied), pindolol, bucindolol, oxprenolol, and alprenolol, which all activate an SCE, were chosen as starting points to correlate the presence of certain functional groups with *β*1-AR SCE activation. To provide molecular explanations for chemical moiety importance, we determined potential SCE-inducing sites for the ligands from modeling and found only one likely site—similar to the G protein-coupled receptor (GPCR) allosteric pocket KS2,^32^ which also overlaps with the pharmacological *β*1-TM4-V189T-L195Q-W199Y site.^25^ From the predicted CGP12177 binding mode, we suggested how certain functional groups interact with KS2 residues, conducted similarity searches for additional *β*-AR ligands, and evaluated them for potential compatibility with KS2. After predicting whether the compounds would/would not bind to KS2 and thus induce an SCE, we pharmacologically evaluated them. Table 8 and Fig. 14 contain a summary of the predictions and results, and we provide a more in-depth reasoning and explanation in the following.

**Table 8:** Summary of compound-*β*1-AR predictions, actual pharmacological outcomes, evidence for SCE activation (A–D) as outlined in the Introduction) from data presented in this study from either CHO-*β*1-luciferase or CHO-*β*1-SPAP cells, and whether the prediction of SCE activation and pharmacological outcome of SCE activation were correct

Compound	Parent Compound	Prediction of Active-State SCE Stabilization	Pharmacological Outcome			Prediction vs Pharmacological Outcome for SCE Activation
			CCE/IBS conformation	SCE conformation	Evidence of SCE activation	
Alprenolol			Agonist (biphasic)	Agonist (biphasic)	C	
Bucindolol			Agonist (biphasic)	Agonist (biphasic)	C	
CGP12177			High-affinity neutral antagonist	Agonist	ABD	
Cimaterol			Agonist	a	None	
Oxprenolol			Agonist (biphasic)	Agonist (biphasic)	C	
Pindolol			Agonist (biphasic)	Agonist (biphasic)	C	
CGP20712A			High-affinity neutral antagonist	Low-affinity neutral antagonist	None	
						
**VL01**	CGP12177	Yes	High-affinity neutral antagonist	Agonist	ABCD	Correct
**VL03** (bunolol)	CGP12177	No	High-affinity neutral antagonist	Low-affinity neutral antagonist	None	Correct
**VL04** (carteolol)	CGP12177	Yes	Agonist (biphasic)	Agonist (biphasic)	ABCD	Correct
**VL05** (moprolol)	Oxprenolol	--	Agonist	a	None	n/a
**VL06**	Bucindolol	Yes	Agonist	a	None	Not correct
**VL07** (propranolol)		No	High-affinity neutral antagonist	Very weak activation	A	Not correct
**VL08**	Alprenolol	No	No receptor interaction	No receptor interaction	None	Correct
**VL09** (bunitrolol)	Alprenolol	No	Agonist (biphasic)	Agonist (biphasic)	ABCD	Not correct
**VL10**	Bucindolol	Yes	Agonist	a	None	Not correct
**VL11**	Bucindolol	Yes	No receptor interaction	No receptor interaction	None	Not correct
**VL12**	Alprenolol	No	No receptor interaction	No receptor interaction	None	Correct
**VL13**	Alprenolol	No	No receptor interaction	No receptor interaction	None	Correct

n/a: prediction not made, this compound was found during similarity searches but its SCE outcome was not predicted ahead of pharmacological studies.

a Although it is not possible to absolutely exclude SCE activation (it may be obscured by significant CCE/IBS activation), the affinity values of CGP20712A suggest inhibition of agonist action at the CCE/IBS only.

**VL03** (bunolol), CGP12177 related, was not predicted to stimulate a *β*1-AR SCE. It lacks bicyclic system nitrogen atoms, rendering it unable to provide hydrogen bond donor functionalities for receptor interactions at KS2, and we would thus have assumed no binding to KS2 and therefore no activation of an SCE. However, **VL03** was a conventional *β*-antagonist—a high-affinity competitive neutral CCE antagonist and a low-affinity competitive neutral antagonist for CGP12177’s SCE. **VL07** (propranolol), predicted not to stimulate a *β*1-AR SCE for the same reason, was a high-affinity CCE neutral antagonist and a low-affinity SCE antagonist in the low-receptor-expressing CHO-*β*1-CRE-luciferase cells (with similar K_D_ values to previous studies^21^). In the high-receptor-expressing cells (CHO-*β*1-CRE-SPAP), where weak agonist responses are more readily detected, **VL07** stimulated a weak partial agonist response. **VL07** (pharmacologically evaluated blind to its identity) yielded K_D_ and EC_50_ values almost identical to previous studies with propranolol.^19^^,^^21^^,^^37^ Also, as previously determined,^19^ the agonist response (log EC_50_ −6.55) differed from its affinity (log K_D_ −8.21 from binding studies and −8.59 from inhibition of cimaterol at the CCE), suggesting that this very weak stimulation is occurring via a *β*1-AR SCE (evidence (A)), in contrast to our prediction.

**VL10**, bucindolol related, has a fluorine rather than the nitrile group. Our prediction suggested the nitrile was not crucial for KS2 binding, and so we would still observe induction of an active-state SCE by **VL10**. **VL10** had lower affinity than bucindolol (−6.73 vs −9.24) and interacted with both the *β*1-AR CCE and CGP12177-induced-SCE (inhibiting both cimaterol and CGP12177 responses), although as with all compounds, with lower SCE affinity. **VL10** stimulated agonist responses that, as expected, were more potent in the higher expressing CHO-*β*1-CRE-SPAP cells (log EC_50_ values −6.81 and −7.73 in the low and high-receptor-expressing cells, respectively), but these responses were readily inhibited by CGP20712A. Furthermore, Fig. 5E suggests **VL10** and cimaterol competition at the same site. Altogether, this suggests that **VL10**’s agonist actions occur via the *β*1-AR CCE. Although an SCE activation at higher **VL10** concentrations cannot be absolutely excluded (it could be obscured by the large CCE agonist response), there is no evidence for SCE activation (compare Figs 3E and 6E with Fig. 5E; **VL10** agonist responses readily inhibited by CGP20712A; agonist response same or left shifted of K_D_). Thus, contrary to our prediction, the nitrile appears important for bucindolol’s SCE activation.

For **VL06**, bucindolol related, with a methoxy group instead of the nitrile, this substitution again reduced affinity and **VL06**’s agonist response was readily inhibited by CGP20712A. Thus, **VL06**, like **VL10**, is a conventional CCE partial agonist. **VL06** and **VL10** indicate that the indole is not important for SCE activation but suggest a role for the nitrile group. **VL11,** bucindolol related, lacking both the nitrile (replaced by a 1-hydroxy-ethoxy group) and the central *β*-hydroxy ether, lost all *β*1-AR interaction, including SCE activation.

**VL05** (moprolol) is oxprenolol related but lacking the terminal alkene moiety. **VL05** agonist responses were monophasic and readily inhibited by CGP20712A, suggesting conventional CCE partial agonism. Thus, oxprenolol’s alkene tail appears important for its stabilization of an active-state SCE.

**VL01**, CGP12177 related, has a double bond replacing one side of the cyclic urea, preserving the second nitrogen as an aromatic amine and hydrogen bond donor (ie, indole instead of 1,3-dihydro-2*H*-benzimidazol-2-one). A previous study suggested the carbonyl group had little pharmacological effect at *β*1-ARs, but the NH group in position 1 (analogous to indole) was essential for active-state SCE induction.^31^ **VL01** was predicted to induce an active-state SCE. **VL01** retained *β*1-AR high affinity (^3^H-CGP12177 binding log K_D_ −9.45, −10.19, and −10.17 from cimaterol response inhibition), suggesting CCE high affinity. **VL01** was a *β*1-AR partial agonist, but the log EC_50_ values (−6.78 (20% isoprenaline) in the low-receptor-expressing cells and −8.56 (70% isoprenaline) in the high-receptor-expressing cells) are considerably different from its CCE high-affinity binding (evidence (A)). **VL01** agonist responses were resistant to CGP20712A inhibition (evidence (B)), and low **VL01** concentrations inhibited cimaterol, distinct from the high concentration required for agonist action (evidence (D), Fig. 7E). **VL01** remained similar to CGP12177—a high-affinity *β*1-AR CCE antagonist with partial agonist activation via an SCE.

**VL04** (carteolol), CGP12177 related, with the urea replaced by an amide positioned to allow receptor interaction, was predicted to stabilize an active-state *β*1-AR SCE. **VL09** (bunitrolol), alprenolol related with a nitrile substituting the *ortho*-propenyl moiety, was predicted not to induce an active-state SCE. Both compounds were high-affinity CCE antagonists, but with right shifted (evidence (A)), antagonist-resistant (evidence (B)) agonist responses in CHO-*β*1-CRE-luciferase cells, suggesting both ligands induce an active-state SCE. In the higher-receptor-expressing CHO-*β*1-CRE-SPAP cells, **VL04** and **VL09** stimulated biphasic responses (evidence (C)). The first component was readily inhibited by CGP20712A, whereas the second component was resistant to antagonism (Fig. 11B), very similar to findings with alprenolol and pindolol.^19^ Thus, both **VL04** and **VL09** activate an SCE.

**VL13** (alprenolol lacking the *ortho*-propylene substituent), **VL08** (alprenolol lacking the *ortho*-propylene, but with 3 methyl groups in *ortho* and *para* positions), and **VL12** (alprenolol without the *β*-hydroxy and a longer aliphatic tail terminating in a methyl-propyl ether instead of the isopropyl group), barely interacted with the *β*1-AR. We predicted these compounds would not lead to an active-state SCE, but they actually lacked affinity or stimulation for either the CCE or SCE. The *β*-hydroxy and *ortho*-propylene groups clearly have important roles, and substituents in the *para* position (even a small methyl) are detrimental.

CGP12177 and pindolol stabilize a shared *β*1-AR active-state SCE via the extracellular end of TM4 (V189-L195-W199), and these residues are within the KS2 site. When mutated to the equivalent *β*2-AR residues (threonine, glutamine, and tyrosine, respectively), the CGP12177 and pindolol active-state SCE is lost (= loss of evidence (A–D) compared with *β*1-WT).^25^ As 3 novel compounds were identified that induce a *β*1-AR active-state SCE, the responses to **VL01**, **VL04,** and **VL09** were therefore examined in the *β*1-V189T-L195Q-M199Y mutant receptor to determine if they were activating the receptor’s SCE via the same site, KS2.

The CCE affinity of ligands measured by ^3^H-CGP12177 binding and inhibition of cimaterol responses were very similar for the wild-type *β*1-AR (*β*1-WT) and *β*1-V189T-L195Q-W199Y receptor, with the exception of ICI118551, where mutation of V189 to threonine increases the ICI118551 affinity.^25^ Cimaterol responses remained similar in *β*1-WT and *β*1-V189T-L195Q-W199Y receptors confirming an unchanged CCE. CGP12177 responses however were more potent (log EC_50_ −9.67 similar to log K_D_ −9.57 = loss of evidence (A)), with lower stimulation (28% rather than 45% of isoprenaline) and readily antagonized by CGP20712A at *β*1-V189T-L195Q-W199Y (= loss of evidence (B)). Thus, CGP12177 appeared as a conventional CCE/IBS partial agonist losing its SCE induction. **VL04** and **VL09’s** biphasic *β*1-WT responses became monophasic at *β*1-V189T-L195Q-W199Y (loss of evidence (C)) with EC_50_ values similar to K_D_ values, lower overall responses (compared with isoprenaline), and responses readily inhibited by CGP20712A. **VL01** had a biphasic response in the *β*1-WT stable mixed cell populations (Fig. 13A, evidence (C)), similar to CGP12177 in some stable mixed populations (eg, Figure 5a of^25^), and suggests that in an amplified system, stabilization of both conformations can be measured. Like CGP12177, **VL01** responses became high potency, monophasic, lower % isoprenaline, and more readily inhibited in the triple mutant (loss of evidence (C)), suggesting a single active-state CCE, and loss of SCE in *β*1-V189T-L195Q-W199Y mutant receptor. Thus, **VL01**, **VL04,** and **VL09** all were unable to induce an active-state SCE in *β*1-V189T-L195Q-W199Y, and their SCE is induced via the same site as by CGP12177 and pindolol—the TM4 site corresponding to KS2.

Thus, 3 more ligands have been identified that stimulate an active-state SCE of the human *β*1-AR, **VL01, VL04,** and **VL09.** Modeling identified only one potential SCE-inducing site and mutagenesis confirmed that **VL01, VL04,** and **VL09** use the same *β*1-TM4-V189T-L195Q-W199Y site as CGP12177 and pindolol. This site corresponds to KS2 from earlier work analyzing a large set of receptors,^32^ which suggested that more class A GPCRs possess a similar cavity. It remains unknown if compounds could be generated that interact with other GPCR KS2 sites and whether SCE inhibition or activation is physiologically relevant.

## Conflict of interest

The authors declare no conflicts of interest.
