# Supplementary material for: Predicting compounds that interact with the 2 known agonist-induced conformations of the human β1-adrenoceptor
Source: Mol Pharmacol. 2025 Oct 1;107(11):100081. doi: 10.1016/j.molpha.2025.100081 (PMC12799587; doi:10.1016/j.molpha.2025.100081)
Supplement: Supplementary Material 1 [file mmc1.pdf]

Predicting compounds that interact with the two known agonist conformations of the human β1-adrenoceptor

Jillian G. Baker[1,2,3,†], Victor Jun Yu Lim[4], Richard G.W. Proudman[1], Franziska N.Z. Giese[4], Peter Kolb[4,†]

[1]Cell Signalling, School of Life Sciences, C Floor Medical School, Queen's Medical Centre, University of Nottingham, Nottingham, NG7 2UH, UK
[2]Respiratory Medicine, Sherwood Forest Hospitals NHS Trust, King's Mill Hospital, Nottinghamshire, UK
[3]Respiratory Medicine, Nottingham University NHS Trust, Queen's Medical Centre, Nottingham, UK
[4]Pharmaceutical Chemistry, Philipps-Universität Marburg, Germany 35037

[†]Corresponding authors
Email: Jillian.Baker@nottingham.ac.uk; peter.kolb@uni-marburg.de
Phone: (+44) 115 8230085; (+49) 6421 28 25908

Supplemental Tables

Supplemental Table 1. SMILES for all compounds mentioned in the text.

**Compound**	**SMILES**
Alprenolol	O(c1ccccc1C\C=C)CC(O)CNC(C)C
Bucindolol	N#Cc0ccccc0OCC(O)CNC(C)(C)Cc1c[nH]c2c1cccc2
CGP12177	CC(C)(C)NCC(COC1=CC=CC2=C1NC(=O)N2)O
Cimaterol	CC(C)NCC(C1=CC(=C(C=C1)N)C#N)O
Oxprenolol	O(c1ccccc1OC\C=C)CC(O)CNC(C)C
Pindolol	CC(C)NCC(O)COc2cccc1[nH]ccc12
VL01	Cc1cc2c(OC[C@@H](O)CNC(C)(C)C)cccc2[nH]1
VL03	CC(C)(C)NC[C@H](O)COc1cccc2c1CCCC2=O
VL04	CC(C)(C)NC[C@@H](O)COc1cccc2c1CCC(=O)N2
VL05	CC(C)NC[C@@H](COC1=CC=CC=C1OC)O
VL06	COC1=CC=C(C=C1)OCC(CNCCC2=CNC3=CC=CC=C32)O
VL07	CC(C)NC[C@@H](COC1=CC=CC2=CC=CC=C21)O
VL08	Cc1cc(C)c(OC[C@@H](O)CNC(C)C)c(C)c1
VL09	CC(C)(C)NC[C@@H](O)COc1ccccc1C#N
VL10	C1=CC=C2C(=C1)C(=CN2)CCNC[C@H](COC3=CC=C(C=C3)F)O
VL11	C1=CC=C2C(=C1)C(=CN2)CCNCC3=CC=C(C=C3)OCCO
VL12	C=CCc1ccccc1OCCNCCCOC
VL13	CC1=CC=C(C=C1)OCC(CNC(C)C)O

Supplemental Table 2. IUPAC names of all compounds mentioned in the text.

**Cmpd**	**IUPAC name**
Alprenolol	(*RS*)-1-(2-allylphenoxy)-3-(isopropylamino)propan-2-ol
Bucindolol	2-[2-hydroxy-3-[[2-(1*H*-indol-3-yl)-1,1-dimethyl-ethyl]amino]propoxy]benzonitrile
CGP12177	4-[3-(tert-butylamino)-2-hydroxypropoxy]-1,3-dihydrobenzimidazol-2-one
Cimaterol	2-amino-5-[1-hydroxy-2-(propan-2-ylamino)ethyl]benzonitrile
Oxprenolol	(*RS*)-1-[2-(allyloxy)phenoxy]-3-(isopropylamino)propan-2-ol
Pindolol	(*RS*)-1-[(1*H*-indol-4-yl)oxy]-3-(isopropylamino)propan-2-ol
VL01	(2*S*)-1-(tert-butylamino)-3-[(2-methyl-1*H*-indol-4-yl)oxy]propan-2-ol
VL03	5-[(2*S*)-3-(tert-butylamino)-2-hydroxypropoxy]-3,4-dihydro-2*H*-naphthalen-1-one
VL04	5-[(2*R*)-3-(tert-butylamino)-2-hydroxypropoxy]-3,4-dihydro-1*H*-quinolin-2-one
VL05	(2*S*)-1-(2-methoxyphenoxy)-3-(propan-2-ylamino)propan-2-ol
VL06	1-[2-(1*H*-indol-3-yl)ethylamino]-3-(4-methoxyphenoxy)propan-2-ol
VL07	(2*S*)-1-naphthalen-1-yloxy-3-(propan-2-ylamino)propan-2-ol
VL08	(2*S*)-1-(propan-2-ylamino)-3-(2,4,6-trimethylphenoxy)propan-2-ol
VL09	2-[(2*R*)-3-(tert-butylamino)-2-hydroxypropoxy]benzonitrile
VL10	(2*R*)-1-(4-fluorophenoxy)-3-[2-(1*H*-indol-3-yl)ethylamino]propan-2-ol
VL11	2-[4-[[2-(1*H*-indol-3-yl)ethylamino]methyl]phenoxy]ethanol
VL12	3-methoxy-*N*-[2-(2-prop-2-enylphenoxy)ethyl]propan-1-amine
VL13	1-(4-methylphenoxy)-3-(propan-2-ylamino)propan-2-ol

Supplemental Captions

b1AR_cgp12177_altconf.pdb: Atomic coordinates of the docking-predicted pose of CGP12177 in KS2.

Swiss_DockCGP.pdb: Atomic coordinates of the multiple poses of CGP12177 generated with Swissdock.
